# Use of the β-Glucan-Producing Lactic Acid Bacteria Strains *Levilactobacillus brevis* and *Pediococcus claussenii* for Sourdough Fermentation—Chemical Characterization and Chemopreventive Potential of In Situ-Enriched Wheat and Rye Sourdoughs and Breads

**DOI:** 10.3390/nu14071510

**Published:** 2022-04-05

**Authors:** Wiebke Schlörmann, Julia A. Bockwoldt, Sabine M. Hübner, Elisa Wittwer, Sarah Reiners, Stefan Lorkowski, Christine Dawczynski, Matthias A. Ehrmann, Michael Glei

**Affiliations:** 1 Department of Applied Nutritional Toxicology, Institute of Nutritional Sciences, Friedrich Schiller University Jena, Dornburger Straße 24, 07743 Jena, Germany; huebner.bine@web.de (S.M.H.); elisa.wittwer@uni-jena.de (E.W.); michael.glei@uni-jena.de (M.G.); 2 Chair of Microbiology, Technical University of Munich, Gregor-Mendel-Straße 4, 85354 Freising, Germany; julia.bockwoldt@tum.de (J.A.B.); matthias.ehrmann@tum.de (M.A.E.); 3 Junior Research Group Nutritional Concepts, Institute of Nutritional Sciences, Friedrich Schiller University Jena, Dornburger Straße 29, 07743 Jena, Germany; sarah.reiners@uni-jena.de (S.R.); christine.dawczynski@uni-jena.de (C.D.); 4 Competence Cluster for Nutrition and Cardiovascular Health (nutriCARD) Halle-Jena-Leipzig, Dornburger Straße 25, 07743 Jena, Germany; stefan.lorkowski@uni-jena.de; 5 Department of Nutritional Biochemistry and Physiology, Institute of Nutritional Sciences, Friedrich Schiller University Jena, Dornburger Straße 25, 07743 Jena, Germany

**Keywords:** β-glucan, colon cancer, chemoprevention, lactic acid bacteria, sourdough, bread

## Abstract

The aim of the present study was to examine β-glucan production and the potential prebiotic and chemopreventive effects of wheat and rye sourdoughs and breads generated with wild-type and non-β-glucan-forming isogenic mutant strains of *Levilactobacillus brevis* and *Pediococcus claussenii*. Sourdough and bread samples were subjected to in vitro digestion and fermentation. Fermentation supernatants (FS) and pellets (FP) were analyzed (pH values, short-chain fatty acids (SCFA), ammonia, bacterial taxa) and the effects of FS on LT97 colon adenoma cell growth, viability, caspase-2 and -3 activity, genotoxic and antigenotoxic effects and on gene and protein expression of *p21*, *cyclin D2*, *catalase* and *superoxide dismutase 2* (*SOD2*) were examined. Concentrations of SCFA were increased and concentrations of ammonia were partly reduced in the FS. The relative abundance of Bifidobacteriaceae was increased in all FPs. Treatment with FS reduced the growth and viability of LT97 cells and significantly increased caspase-2 and -3 activities without exhibiting genotoxic or antigenotoxic effects. The *p21* mRNA and protein levels were increased while that of *cyclin D2* was reduced. *Catalase* and *SOD2* mRNA and protein expression were marginally induced. The presented results indicate a comparable chemopreventive potential of wheat and rye sourdoughs and breads without an additional effect of the formed β-glucan.

## 1. Introduction

The use of lactic acid bacteria (LAB) as starter cultures in sourdough fermentation has a long tradition in the production of baked goods such as wheat or rye breads. The main advantages of the use of sourdoughs and LAB as starters in the fermentation process are the positive effects on shelf-life and structural as well as sensory properties of the resulting breads [1,2,3]. During sourdough fermentation, the LAB form exopolysaccharides (EPS), which improve the water holding capacity and rheological properties of the doughs as well as the texture of the resulting breads [1,4,5,6]. Furthermore, the high-molecular-weight EPS may also improve the nutritional value of the breads. Results from in vitro and in vivo studies indicate an association between EPS and health-promoting properties, such as prebiotic, immunomodulatory, antioxidant, anticancer and cholesterol-lowering potential [6,7,8]. EPS are formed by different pathways resulting in homopolysaccharides (HoPS), which consist of one type of monosaccharide (glucose, fructose) and heteropolysaccharides (HePS) consisting of two or more different monosaccharides (e.g., glucose, galactose, rhamnose) in repeating units. The extracellular synthesis of HoPS depends on sucrose as substrate and, in general, results in higher yields than the synthesis of HePS, which includes the intracellular synthesis of sugar nucleotide precursors and the extracellular polymerization by glycosyltransferases [7,8,9]. An exception is the synthesis of β-glucan by LAB, such as the wine- and beer-spoiling strains of *Levilactobacillus* (*L.*) *brevis* and *Pediococcus (P.) claussenii*. Though β-glucan is a HoPS, its synthesis is similar to that of HePS. The brewery isolates *L. brevis* TMW (Technische Mikrobiologie Weihenstephan) 1.2112 [10] and *P. claussenii* TMW 2.340, for example, form this HoPS as capsular β-(1,3)-glucan with β-(1,2)-branches around the cells [11]. The advantages of using β-glucan-producing LAB as starter for sourdough fermentation are the structure-forming effects of the network-like capsular β-glucan, such as improving the rheological properties and increasing the viscosity of the doughs, already in relatively low amounts. Furthermore, no addition of sucrose is required for the formation of β-glucan [12,13]. In particular, the use of β-glucan-producing LAB represents a promising alternative to naturally enrich breads with β-glucan, not only to improve the rheological and sensory properties of the doughs and the resulting breads but also to improve their nutritional value. In general, results from several studies indicate that β-glucans from different sources exert positive health effects, such as prebiotic, anticancer, anti-inflammatory, and immunomodulatory effects [14]. In particular, for oat and barley β-glucan, health-promoting physiological properties, such as the reduction in plasma cholesterol levels and glycemic response modulating effects were described, and a regular intake of oat and barley is associated with a reduced risk for developing cardiovascular disease or diabetes [15,16,17,18]. In vitro studies also indicate a chemopreventive potential [19,20,21,22]. Furthermore, for LAB β-glucans or β-glucan-producing LAB, probiotic [23], prebiotic [24] and immunomodulatory potential [25] have been described. In a recent in vitro study, we demonstrated prebiotic, cholesterol-reducing and chemopreventive effects of isolated LAB β-glucans [26]. Nevertheless, until now, similar effects of LAB β-glucans as a natural network- and a structure-forming component of the complex food matrix in sourdoughs and breads have not yet been studied. Therefore, the aim of the present study was to investigate potential health-promoting properties regarding the fermentation profile and potential prebiotic as well as the chemopreventive effects of wheat and rye sourdoughs and breads obtained after fermentation with *L. brevis* TMW 1.2112 and *P. claussenii* TMW 2.340. Furthermore, the health-related effects of these in situ-enriched sourdoughs and breads and their nutrient composition and β-glucan content were compared to that of sourdoughs and breads obtained after fermentation with the respective non-β-glucan-forming mutant strains (Δ*gtf*-2) *L. brevis* TMW 1.2320 and *P. claussenii* TMW 2.2123.

## 2. Materials and Methods

### 2.1. Cultivation of L. brevis and P. claussenii Strains and Preparation of Sourdoughs and Breads

*L. brevis* TMW 1.2112 and *P. claussenii* TMW 2.340 as well as the two non-β-glucan-forming mutant strains Δ*gtf*-2 *L. brevis* TMW 1.2320 and *P. claussenii* TMW 2.2123 were cultivated overnight in modified de Man, Rogosa and Sharpe medium (mMRS), under static conditions at 30 °C. The mMRS medium (pH 6.2) was composed of (quantities per liter): 10 g of peptone, 5 g of yeast extract, 5 g of meat extract, 4 g of K_2_HPO_4_ · 3H_2_O, 2.6 g of KH_2_PO_4_, 3 g of NH_4_Cl, 1 g of Tween^®^-80, 0.5 g of cysteine-HCl, 20 g of maltose, 0.2 g of MgSO_4_·7H_2_O and 0.038 g of MnSO_4_·H_2_O. The inoculation and fermentation of the wheat and rye sourdoughs and preparation of the breads were performed as previously described by Bockwoldt et al. [12]. The sourdoughs were fermented for 24 h at 28 °C with dough yields of 200. After freezing at −80 °C, the dough and bread samples were freeze-dried (Freezone 2.5, Labconco Corporation, Kansas City, MO, USA) and used for further analysis. The following chemicals were purchased from Carl Roth GmbH & Co. KG (Karlsruhe, Germany): Peptone, yeast extract, NH_4_Cl, cysteine and MnSO_4_·H_2_O; GERBU Biotechnik GmbH (Heidelberg, Germany): maltose; Merck KGaA (Darmstadt, Germany): meat extract, Tween^®^-80; VWR International (Radnor, PA, USA): K_2_HPO_4_· 3H_2_O, KH_2_PO_4_; Sigma-Aldrich (St. Louis, MO, USA): MnSO_4_·H_2_O.

### 2.2. Quantification of Main Nutrients in Sourdoughs and Breads

Wheat and rye sourdoughs, as well as the breads obtained after fermentation with LAB starters, were analyzed as freeze-dried powders. All quantifications of the main constituents were carried out in duplicate and in accordance with the official methods of the Association of Official Agricultural Chemists (AOAC) [27].

#### 2.2.1. Dietary Fiber

The total dietary fiber content was analyzed enzymatically and gravimetrically with the BIOQUANT^®^ Total Dietary Fiber kit (Merck, Darmstadt, Germany) according to Lee et al. [28] using two duplicate samples for protein and ash content determination. The protein contents of resulting residues were determined using the Kjeldahl method [29] and the quantification of the ash content was performed after heating in a muffle furnace at 525 °C. The mean weight of the two residues after subtraction of the protein and ash values corresponds to the content of dietary fiber in the sample.

#### 2.2.2. Crude Protein

For determination of the crude protein content, the nitrogen content was quantified using the Kjeldahl method [29] according to DIN EN ISO 20483:2014-03, using approximately 1.5 g of the samples. The protein contents of the samples were determined using a nitrogen to protein conversion factor of 5.7 for wheat and rye products (DIN EN ISO 20483:2014-03).

#### 2.2.3. Crude Fat

Total fat content in the samples was determined using the Soxhlet method combined with acid hydrolysis of the Weibull–Stoldt method [30].

### 2.3. Quantification of Bacterial β-Glucan in Sourdoughs and Breads

The in situ-produced bacterial β-glucan within the fermented doughs and the sourdough breads were quantified by a competitive enzyme-linked immuno-sorbent assay (ELISA), as previously described by Werning et al. [31] and modified according to Bockwoldt et al. [12]. Beta-glucan of *L. brevis* TMW 1.2112 was used for this assay and therefore the strain was cultivated in a semi-defined medium [32] with modifications [12] for 30 h at 30 °C. The supernatant was separated from the cells by centrifugation (16,000× *g*, 4 °C for 30 min), the pellet was discarded, and the supernatant precipitated with ice-cold ethanol (three-fold) (VWR International, Radnor, PA, USA) overnight at 4 °C [33]. The next steps were the collection of precipitated β-glucan by centrifugation (10,000× *g*, 4 °C for 15 min), and the dissolving of the precipitate in distilled water. To reduce remaining protein impurities, the solution was precipitated with perchloric acid (Sigma-Aldrich Inc., St. Louis, MO, USA) and centrifuged. The resulting supernatant was dialyzed and freeze-dried [12].

### 2.4. In Vitro Digestion and Fermentation

The in vitro digestion and fermentation experiments were performed as described earlier [20]. According to Barry et al. [34], 10 g/L fermentable substance of freeze-dried wheat and rye sourdoughs and bread samples were used equivalently to 0.5 g of the nearly completely fermentable positive control Synergy1^®^ (Orafti^®^Synergy1, Beneo, Mannheim, Germany). A batch without a fermentable substance (blank) served as the fermentation negative control. In brief, samples were resuspended in potassium phosphate buffer (0.1 M, pH 7.0, KH_2_PO_4_, Carl Roth GmbH & Co. KG, Karlsruhe, Germany) followed by treatments to simulate the different stages of digestion (mouth, stomach, small and large intestine). For the simulation of the conditions in the large intestine, pre-digested samples were mixed with equal proportions of a feces inoculum mixture obtained from at least three healthy donors and the pH values of the samples were adjusted to 6.5. In vitro fermentation was performed under anaerobic conditions for 24 h. All steps of the simulated digestion were carried out at 37 °C in a shaking water bath. The fermentation process was stopped on ice after measuring the pH values of each sample. After centrifugation, fermentation supernatants (FS) and pellets (FP) were stored at −80 °C until use. FS were sterile filtered (Filtropur S, 0.22 µm, Sarstedt AG & Co. KG, Nürnbrecht, Germany) prior to usage.

The feces collection was approved by the Ethics Committee of the University Hospital of the Friedrich Schiller University Jena, Germany (No. 4625-12/15). Written informed consent was obtained from all subjects.

### 2.5. Determination of Short-Chain Fatty Acid Concentrations

Concentrations of short-chain fatty acids (SCFA) in FS were measured in duplicate by flame ionization detector (FID)-coupled gas chromatography (GC-FID) as described [35] using iso-caproic acid (Sigma-Aldrich Inc., St. Louis, MO, USA) in concentrated formic acid (Sigma-Aldrich Inc., St. Louis, MO, USA) as internal standard. For the GC-FID measurement (GC-17A V3 equipped with an autosampler AOC-5000 and an FID, Shimadzu, Kyoto, Japan), 1 µL of the mixture was used for injection. SCFA were separated on a Zebrone FFAP capillary GC column (15 m, Phenomenex, CA, USA).

### 2.6. Determination of Ammonia Concentrations

Ammonia concentrations in FS were measured as described previously [20] by adding 1% (*w*/*v*) phenol (Fluka Biochemika, Buchs, Switzerland) and 0.005% (*m*/*v*) sodium nitroprusside (Carl Roth GmbH & Co. KG, Karlsruhe, Germany) (in aqua bidest.) and a 0.5% NaOH (*w*/*v*) (Carl Roth GmbH & Co. KG, Karlsruhe, Germany) and 0.95% (*v*/*v*) sodium hypochlorite solution (Sigma-Aldrich Inc., St. Louis, MO, USA) (in aqua bidest). Samples were heated to 60 °C for 10 min, cooled down to room temperature, and 200 µL of the samples were transferred to a 96-well plate. Extinction was measured in triplicate at λ = 630 nm (GENios, Tecan Germany, Crailsheim, Germany) and ammonia concentrations in the FS were calculated based on the standard dilution series of ammonia chloride (0–10 mM) (Carl Roth GmbH & Co. KG, Karlsruhe, Germany).

### 2.7. Quantification of Bacteria Species via 16SrDNA Amplicon Sequencing of Fermentation Pellets

Bacteria species in FP of the different sourdough and bread preparations were analyzed by 16S rDNA sequencing, amplifying the V3/V4 regions using the UPARSE-based IMNGS (integrated microbial next-generation sequencing) platform [36,37]. A sample without template DNA served as the negative control. A de-multiplexing (demultiplexer v3.pl) was performed before the sequences were trimmed by ten nucleotides and a quality score of unpaired reads of three. Sequences with nucleotides <200 and >600 and expected errors in paired reads >3 were excluded and samples were screened for chimeras [38]. Operational taxonomic units (OTUs) were clustered at 97% similarity and OTUs with a relative abundance of <0.25% were removed. To generate a graphical overview of the microbial composition, taxonomic binning was performed by Rhea using the set of R-scripts [39].

### 2.8. Cell Culture

Cell culture experiments were performed with the human colorectal adenoma cell line LT97 (a kind gift from Professor B. Marian, Institute for Cancer Research, University of Vienna, Vienna, Austria). This cell line was prepared from colon microadenoma of a patient suffering from hereditary familiar polyposis coli [40] and therefore represents a model of an early stage of colon carcinogenesis. The composition of the cell culture medium, as well as culture conditions, was already described in detail elsewhere [41]. For the treatment of cells, FS were diluted in cell culture medium to final concentrations.

### 2.9. DAPI Assay

Growth inhibitory effects of FS of the different sourdough and bread preparations on LT97 colon adenoma cells were analyzed using the DAPI (4′,6-diamidino-2-phenylindol) assay, as described earlier [42]. Relative cell numbers were calculated from the blank-corrected results based on the medium control, which was set to 100%. A nonlinear regression (one-phase exponential decay, GraphPad Prism^®^ version 5, GraphPad Software, San Diego, CA, USA) was performed to calculate sub-toxic concentrations of FS (2.5% and 5%) below EC_50_ for further cell culture experiments.

### 2.10. MTT Assay

The MTT assay was performed according to Mosmann [43] and Wang et al. [44] with slight modifications. After the treatment of LT97 cells with different concentrations of FS obtained from sourdoughs and breads (2.5%, 5%, 10% and 20%) for 24 h, 48 h and 72 h, respectively, the incubation mixtures were replaced by 100 µL fresh medium and 10 µL of MTT reagent (3-(4,5-dimethylthiazol-2-yl)-2,5-diphenyl tetrazolium bromide, 50 mg in 10 mL PBS, Sigma-Aldrich Inc., St. Louis, MO, USA) was added to the cells. After treatment for 3 h at 37 °C, 200 µL DMSO (dimethyl sulfoxide, Carl Roth GmbH & Co. KG, Karlsruhe, Germany) was added and after 5 min and resuspension, absorbance was measured at 570 and 630 nm (Synergy 2, BioTek, Bad Friedrichshall, Germany). Results were calculated from the blank-corrected values based on the medium control, which was set to 100%.

### 2.11. Comet Assay

Potential genotoxic and antigenotoxic effects of FS obtained from the different sourdough and bread samples were examined with the alkaline version of the Comet assay, as described [45] with minor modifications. To analyze short-term effects, LT97 cells were grown in 75 cm^2^ cell culture flasks to a confluence of 80–90% and, after harvesting, 0.1 × 10^6^ cells were treated with 2.5% and 5% FS (in PBS, phosphate-buffered saline, pH 7.4) for 1 h (genotoxic effects) as well as for 45 min following 15 min co-incubation with 75 µM H_2_O_2_ (Merck KGaA, Darmstadt, Germany) (antigenotoxic effects).

For the analysis of long-term effects, cells were grown in 6-well plates to a confluence of 50–60% and incubated with 2.5% and 5% FS (in cell culture medium) for 24 h. Subsequently, cells were washed with PBS, harvested and adjusted to a number of 0.1 × 10^6^ cells to examine potential genotoxic effects. Potential antigenotoxic effects were investigated with 0.1 × 10^6^ cells treated with 75 µM H_2_O_2_ for an additional 15 min. All cell treatments were carried out at 37 °C in an incubator with 5% CO_2_ and 95% humidity. Subsequently, cells were washed with PBS and after centrifugation (425× *g*, 5 min, 4 °C) cell pellets were stored on ice until they were mixed with 45 μL 0.7% low-melting agarose (Biozym, Hessisch Oldendorf, Germany) and spread onto pre-coated (0.5% normal melting agarose, Biozym, Hessisch Oldendorf, Germany) microscopic slides. Cells treated with H_2_O_2_ (75 μM, 15 min) and PBS were used as the positive and negative control, respectively. The Comet assay and detection of DNA damage (% TI, tail intensity; means of 100 counted cells) was performed as described [45].

### 2.12. Caspase Assay

To analyze the growth inhibitory effects of the FS obtained from sourdoughs and breads in more detail, the induction of caspase-2 and -3 was determined as a marker of apoptosis in LT97 cells as described previously [26] with slight modifications. LT97 cells were grown in 6-well plates and treated with 2.5% and 5% FS for 24 h or 48 h. Cells treated with medium and butyrate (4 mM, Na-butyrate, Merck KGaA, Darmstadt, Germany) served as a negative and positive control, respectively. After treatment with FS and controls, prepared cell lysates [26] were treated with 50 nM caspase inhibitors (caspase-3: Ac-DEVD-CHO; caspase-2: Ac-VDVAD-CHO, Enzo Life Science, Lörrach, Germany) for 10 min (4 °C) and/or with 25 µM caspase substrate (caspase-3: Ac-DEVD-AMC, caspase-2: Ac-VDVAD-AMC, Enzo Life Science) for 2 h (37 °C) in 96-well plates. Subsequently, fluorescence was measured at Ex/Em λ = 380/465 nm (SpectraFluor Plus, Tecan Germany, Crailsheim, Germany) and the modulation of caspase activity was calculated relative to the medium control, which was set to 1 after the subtraction of signals obtained from treatment with caspase inhibitor and the substrate negative control. HEPES, CHAPS, DTT, leupeptin, pefabloc and PMSF were purchased from Carl Roth GmbH & Co. KG (Karlsruhe, Germany) and pepstatin A and sodium orthovanadate were purchased from Sigma-Aldrich Inc. (St. Louis, MO, USA).

### 2.13. Analyses of mRNA Expression by qPCR

#### 2.13.1. Isolation of Total RNA

LT97 cells were grown in 6-well plates to a confluence of 50–60% until they were treated with 2.5% and 5% FS obtained from sourdoughs and breads as well as from controls (blank, Synergy1^®^ and 4 mM butyrate). After 6 h and 24 h of treatment, cells were washed with PBS, harvested with trypsin/EDTA (ethylene diamine tetra-acetic acid, Sigma-Aldrich Chemie GmbH, Taufkirchen, Germany), and total RNA was isolated using the NucleoSpin^®^ RNA Plus Kit (Machery–Nagel, Düren, Germany) according to the manufacturer’s instructions. The concentration and quality, as well as the integrity of isolated RNA, were analyzed using the NanoDrop^®^ (PEQLAB Biotechnologie GmbH, Erlangen, Germany) as well as the Agilent RNA 6000 Nano Kit and the Agilent 2100 Bioanalyzer (Agilent Technologies, Waldbronn, Germany), respectively, according to the manufacturer’s instructions. Only RNA samples with an RNA integrity number >9 were used for further experiments.

#### 2.13.2. Synthesis of cDNA

For the synthesis of cDNA from total RNA, the SCRIPT cDNA Synthesis Kit (Jena Bioscience GmbH, Jena, Germany) was used. For this, 1.5 µg RNA was diluted with RNase-free water to a volume of 11.5 µL. After adding 1 µL Oligo(dT)_15_-Primer (100 µM, Jena Bioscience GmbH, Jena, Germany), denaturation was performed for 5 min at 65 °C. Subsequently, samples were cooled on ice for 1 min and 7.5 µL of a reaction mix containing 5 × reaction buffer, 0.1 M DTT, 10 mM dNTPs, and 200 U/µL reverse transcriptases were added. Transcription was performed at 42 °C for 50 min and the reaction was stopped at 70 °C for 15 min. Non-transcribed RNA was degraded by a final treatment with RNase H (2.5 U/µL, New England Biolabs GmbH, Frankfurt am Main, Germany) at 37 °C for 20 min.

##### 2.13.3. qPCR Experiments

Analysis of the relative mRNA expression of the target genes *catalase*, *SOD2*, *p21* and *cyclin D2* was performed by quantitative real-time PCR (qPCR) using the CFX Connect Real-Time PCR Detection System (BioRad Laboratories, Feldkirchen, Germany). For this, cDNA samples were diluted at 1:50 in RNase-free water and qPCR experiments were conducted in duplicate in a 10 µL reaction mix using the GoTaq^®^ qPCR Master Mix (Promega, Mannheim, Germany) (*SOD2* and *cyclin D2*), iTaq™ Universal SYBR^®^ Green Supermix (BioRad Laboratories, Feldkirchen, Germany) (*catalase*), SsoAdvanced™ Universal SYBR^®^ Green Supermix (BioRad Laboratories, Feldkirchen, Germany) (*p21*), RNase-free water and 10 pmol of gene-specific primers (Eurofins Genomics, Ebersberg, Germany) (Table 1). The qPCR analyses of *SOD2*, *catalase* and *cyclin D2* included the following steps: initial denaturation (95 °C, 2 min), 40 cycles of denaturation (94 °C, 15 s), annealing (58 °C, 15 s) and extension (72 °C, 20 s), followed by a final extension step (72 °C, 10 s) and melting-curve analysis (65–95 °C, 5 s, 70 cycles). The qPCR for *p21* included an initial denaturation (95 °C, 2 min), 40 cycles of denaturation (94 °C, 30 s), annealing (58 °C, 30 s) and extension (72 °C, 40 s), followed by the final extension (72 °C, 10 min) and melting-curve analysis (65–95 °C, 5 s, 70 cycles). The relative mRNA expression of the target genes was normalized to the geometric mean of the expression of the two reference genes *β-actin* and *GAPDH* based on the equation of Pfaffl et al. [46]. Data were log_2_-transformed prior to statistical analysis [47].

### 2.14. Analyses of Protein Expression by Western Blotting

Protein expression of p21 and cyclin D2, as well as of catalase and SOD2, was measured via SDS-PAGE and Western blot analyses. For this, LT97 cells were treated with FS obtained from sourdoughs and breads as well as from controls (blank, Synergy1^®^ and butyrate (4 mM)) for 24 h and 48 h. Then, cells in the supernatant and from the wells were collected and homogenized in lysis buffer (10 mM Tris/HCl pH 8.0, 75 mM NaCl, 5% glycerol, 1 mM EDTA, 1 mM DDT, 1 mM sodium orthovanadate, 1% Nonidet P40, Protease inhibitor mix (Pierce^TM^ Protease Inhibitor Mini Tablets, Thermo Scientific, Rockford, IL, USA)) and incubated on ice for 10 min. Homogenates were centrifuged (20 min, 16,000× *g*, 4 °C) and total protein concentrations were measured using the method of Bradford [48]. For separation via SDS-PAGE, 25 µg protein was reconstituted in 4× concentrated loading buffer (0.25 M Tris pH 6.8, 8% SDS, 40% glycerol, 20% 2-β-mercaptoethanol, 0.01% Orange G) and denatured for 5 min at 95 °C. A 5% stacking gel (0.125 M Tris/HCl pH 8.8, 6% acrylamide, 0.1% SDS, 0.1% ammonium persulfate, 0.1% *N*,*N*,*N*’,*N*’-tetramethylethylenediamine, TEMED) and 12% separation gel (0.375 M Tris/HCl pH 6.8, 12% acrylamide, 0.1% SDS, 0.1% ammonium persulfate and 0.04% TEMED) were prepared and SDS-PAGE was run for 1.5 h at 100 V in electrophoresis buffer (50 mM Tris/HCl pH 8.5, 0.38 M glycine, 0.1% SDS). Subsequently, Western blotting was performed by blotting the proteins onto nitrocellulose membranes (Thermo Scientific, Waltham, MA, USA) using the Bio-Rad Trans-Blot Turbo System (BioRad Laboratories, Munich, Germany). Unspecific binding sites were blocked by treatment with Odyssey Blocking Buffer (1:1 in PBS, LI-COR Biosciences GmbH, Bad Homburg, Germany) for 1 h. Membranes were incubated with the following primary antibodies: mouse anti-p21 (1:500, Cell Signaling Technology, Beverly, MA, USA), rabbit anti-cyclin D2 (1:1000) (Cell Signaling Technology, Beverly, MA, USA), mouse anti-catalase (1:1000, Abnova, Taipei, Taiwan), mouse anti-SOD2 (1:500–1000, OriGene Technologies, Inc., Rockville, MD, USA), rabbit anti-GAPDH (1:5000, Abcam, Cambridge, UK), mouse-anti-GAPDH (1:5000, Abcam, Cambridge, UK) overnight (4 °C). Treatment with the following secondary antibodies was performed for 1 h at room temperature: IRDye 800 CW donkey anti-mouse IgG, IRDye 680 LT goat anti-rabbit IgG, IRDye 680 CW donkey anti-mouse IgG, IRDye 800 LT goat anti-rabbit IgG (1:15,000) (LI-COR Biosciences GmbH, Bad Homburg). Protein bands were visualized and quantified using the Odyssey system (LI-COR Biosciences GmbH, Bad Homburg, Germany). Results are expressed as fold changes in relation to the medium control, which was set to 1. Unless stated otherwise, chemicals were purchased from Carl Roth GmbH & Co. KG (Karlsruhe, Germany) except sodium orthovanadate, 2-β-mercaptoethanol, and glycine (Sigma-Aldrich Inc. St. Louis, MO, USA) as well as Nonidet P40 (Roche Diagnostics GmbH, Mannheim, Germany).

### 2.15. Statistical Analyses

Unless stated otherwise, means and standard deviations of at least three independent experiments were calculated and statistical differences were analyzed by one-way or two-way ANOVA and Ryan–Einot–Gabriel–Welsh F post hoc test. The unpaired Student’s *t*-test was used for the comparison of the two groups. Statistical analyses were performed using the SPSS Statistics software, version 25 (IBM Corporation, Armonk, NY, USA).

## 3. Results

### 3.1. Quantification of Main Nutrients in Sourdoughs and Breads

The contents of total dietary fiber, protein, fat and ash were measured in samples of freeze-dried wheat and rye sourdoughs and the respective bread samples that were obtained by fermentation using the β-glucan-producing LAB *P. claussenii* and *L. brevis* as starters. In addition, the sourdoughs and breads obtained after fermentation with the non-β-glucan-producing mutant strains of the respective LAB were analyzed (Table 2). Contents of the total dietary fiber of wheat and rye sourdoughs and breads obtained from fermentation with the wild-type strain of *P. claussenii* were higher than in wheat and rye sourdoughs and breads after fermentation with the respective mutant strain. In general, higher dietary fiber contents were measured in the breads obtained from *P. claussenii* sourdough fermentation in comparison to sourdoughs. No differences in the dietary fiber content were observed between the sourdoughs or breads obtained after fermentation with *L. brevis* wild-type or mutant strains. In general, dietary fiber contents were higher in rye than in wheat samples. In contrast, amounts of protein were higher in wheat than in rye samples. Overall, the protein contents were similar between the sourdoughs and breads obtained from fermentations with the different LAB starters and the fat contents were very similar, too. Ash contents tended to be lower in wheat and rye sourdoughs compared to wheat and rye breads. Taken together, the main constituents did not differ markedly between samples obtained after fermentation with wild-type or mutant strains, except for the dietary fiber content in samples obtained from *P. claussenii* fermentation.

### 3.2. Quantification of β-Glucan in Sourdoughs and Breads

Contents of β-glucan were markedly higher in wheat and rye sourdoughs and breads obtained after fermentation with the wild-type strains of *P. claussenii* and *L. brevis* (100.2–674.2 µg/g) than after fermentation with the respective mutant strains (0.0–40.3 µg/g) (Table 2).

### 3.3. Characterization of Fermentation Samples Obtained from Sourdoughs and Breads

#### 3.3.1. pH Values

After in vitro fermentation, pH values of the FS obtained from the fermentation positive control Synergy1^®^ (pH 5.2) and wheat, as well as rye sourdoughs (pH 5.4, on average), were markedly lower than the pH measured in the FS of the fermentation negative control (blank, pH 6.7) (Table 3). Similar results were obtained for bread samples obtained from wheat and rye sourdough fermentation with the different LAB starters (Table 4).

#### 3.3.2. Concentrations of SCFA

Compared to the blank FS, concentrations of the main SCFA (acetate, propionate and butyrate) increased upon fermentation of Synergy1^®^ (2.7-fold). A similar increase in SCFA concentrations of 2.5-fold was observed in the FS of wheat and rye sourdoughs (Table 3). Comparable results were obtained after in vitro fermentation of wheat and rye breads produced from LAB sourdoughs (Table 4). Again, SCFA concentrations in FS of wheat and rye breads were 2.5-fold higher than the SCFA concentration in the blank FS and a similar increase was observed for the FS of Synergy1^®^ (2.4-fold). Compared to the molar SCFA ratios (acetate:propionate:butyrate) determined for the blank FS, molar ratios shifted towards butyrate in FS of LAB-fermented wheat and rye sourdoughs (Table 3). These were comparable with the ratio calculated for the FS of Synergy1^®^. In comparison, in vitro fermentation of wheat and rye breads produced from LAB sourdoughs also resulted in a shift of molar ratios towards butyrate production, but to a lesser extent (Table 4). In general, no differences regarding pH values and SCFA concentrations, as well as molar ratios of SCFA, were observed between wheat and rye sourdoughs and breads obtained from fermentations with the different wild-type or mutant LAB strains of *P. claussenii* and *L. brevis*.

#### 3.3.3. Concentrations of Ammonia

After in vitro fermentation, ammonia concentration was significantly lower in the FS of the fermentation positive control Synergy1^®^ than in the FS of the blank control (Table 3). Significantly lower ammonia concentrations were also measured in the FS of wheat sourdoughs obtained after fermentation with *P. claussenii* wild-type and mutant strain, while ammonia concentrations were decreased insignificantly in the FS of wheat sourdoughs obtained after fermentation with *L. brevis* wild-type and mutant strain. FS of rye sourdoughs produced with wild-type and mutant strains of *P. claussenii* and *L. brevis* also contained significantly lower ammonia concentrations than the blank FS. No significant differences between ammonia concentrations in the blank FS and in the FS obtained from wheat and rye breads produced with the different LAB starters were observed (Table 4). In contrast, the FS of Synergy1^®^ contained significantly lower amounts of ammonia. In general, no differences regarding ammonia concentrations were observed between wheat and rye sourdoughs and breads obtained from fermentations with the different wild-type or mutant LAB strains of *P. claussenii* and *L. brevis*.

### 3.4. Modulation of Bacteria Species in FP

**Figure 1:**
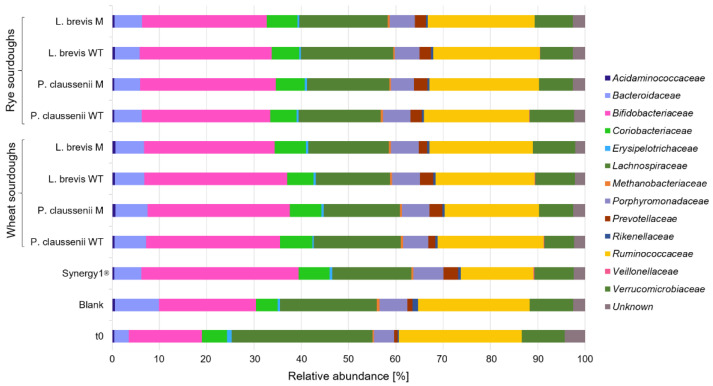
Relative abundance [%] of bacterial families in fermentation pellets (FP) of controls (blank, Synergy1^®^) and in FP of wheat and rye sourdoughs obtained after sourdough fermentation with wild-type (WT) and mutant (M) strains of *Pediococcus claussenii* (WT: TMW 2.340, M: TMW 2.2123) and *Levilactobacillus brevis* (WT: TMW 1.2112, M: TMW 1.2320). TMW: Technische Mikrobiologie Weihenstephan.

**Figure 2:**
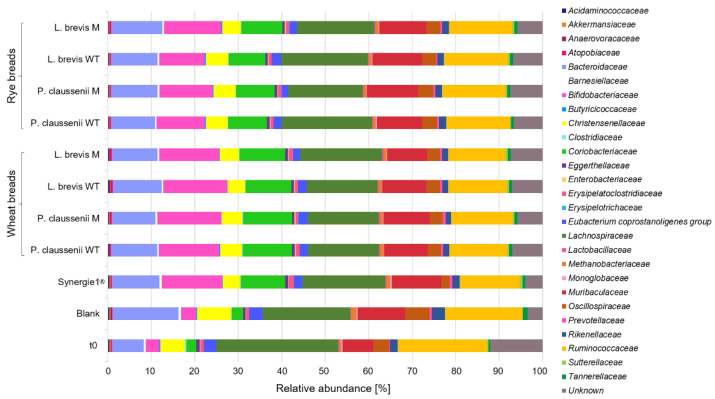
Relative abundance [%] of bacterial families in fermentation pellets (FP) of controls (blank, Synergy1^®^) and in FP of wheat and rye breads obtained after sourdough fermentation with wild-type (WT) and mutant (M) strains of *Pediococcus claussenii* (WT: TMW 2.340, M: TMW 2.2123) and *Levilactobacillus brevis* (WT: TMW 1.2112, M: TMW 1.2320). TMW: Technische Mikrobiologie Weihenstephan.

In general, bacteria species were modulated differentially during the in vitro fermentations of wheat and rye sourdoughs and breads. Most obvious, the fermentation of the sourdough samples and controls resulted in lower counts of different bacteria families (Figure 1) compared to the in vitro fermentation series of the bread samples, including controls (Figure 2). Furthermore, the most pronounced differences regarding the bacteria composition were observed between the pure feces samples at time point t0 before the in vitro fermentation experiments and samples, including the blank controls, after 24 h of in vitro fermentation (Figure 1 and Figure 2, Supplemental Table S1). Proportions of *Bifidobacteriaceae* were markedly higher in FP of Synergy1^®^ and in FP of wheat and rye sourdoughs than in the FP of the blank control. Higher levels of *Bifidobacteriaceae* were also detected in FP of Synergy1^®^ as well as wheat and rye breads than in the FP of the respective blank control. Compared to the blank control, an increase of bacteria was also detected for *Coriobacteriaceae* in FP of Synergy^®^ and in the FP of wheat and rye sourdoughs. This increase was more pronounced in the fermentation series of the bread samples. Compared to the time point t0 of both fermentation series of sourdoughs and breads, the relative levels of *Bacteroidaceae* increased during fermentation in the blank controls and declined again in FP of sourdoughs and breads. A decrease of bacteria families was also observed for *Lachnospiraceae* and *Ruminococcaceae* during the fermentation of wheat and rye sourdoughs and breads.

In general, no differences regarding bacteria composition were observed between FP obtained from LAB wild-type- or mutant-generated sourdoughs and breads, whereas marginal differences were detected between FP obtained from wheat and rye samples.

### 3.5. Cytotoxic Effects of FS

**Figure 3:**
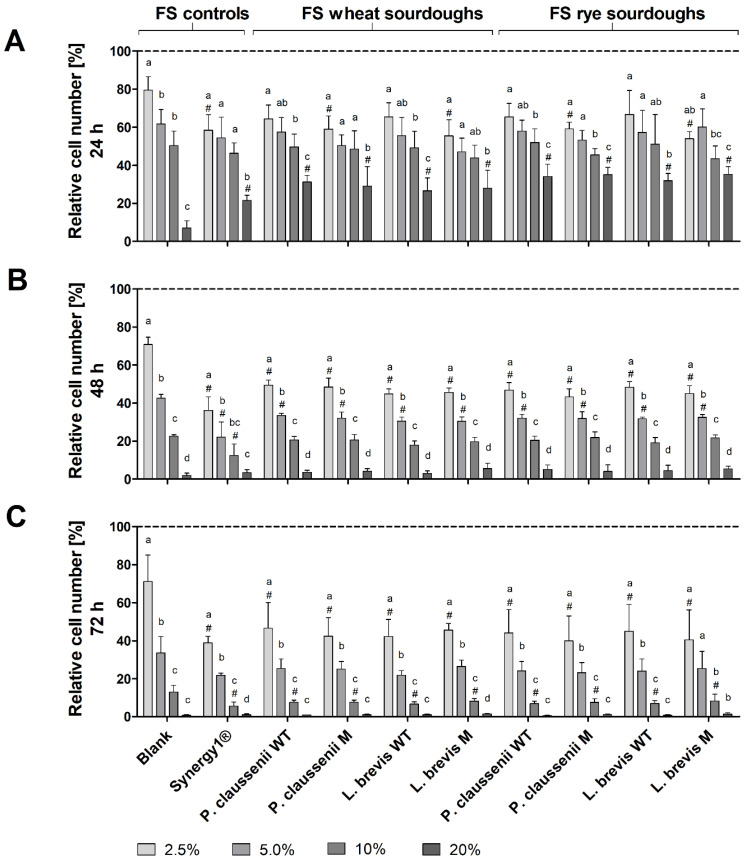
Relative number [%] of LT97 colon adenoma cells after treatment with different concentrations (2.5%, 5%, 10% and 20%) of fermentation supernatants (FS) obtained from controls (blank, Synergy1^®^) and wheat and rye sourdoughs for 24 h (**A**), 48 h (**B**) and 72 h (**C**). Sourdoughs were generated using wild-type (WT) and mutant (M) strains of *Pediococcus claussenii* (WT: TMW 2.340, M: TMW 2.2123) and *Levilactobacillus brevis* (WT: TMW 1.2112, M: TMW 1.2320). Results were calculated on the basis of the medium control (dashed line), which was set at 100% (mean + SD, *n* = 4). Significant differences between cells treated with FS blank and cells treated with FS of all other samples (^#^ *p* < 0.05) and significant differences between cells treated with different concentrations of FS (^a–d^ *p* < 0.05, different letters represent significantly different results) were obtained by one-way ANOVA and F-test according to Ryan–Einot–Gabriel–Welsh. TMW: Technische Mikrobiologie Weihenstephan.

**Figure 4:**
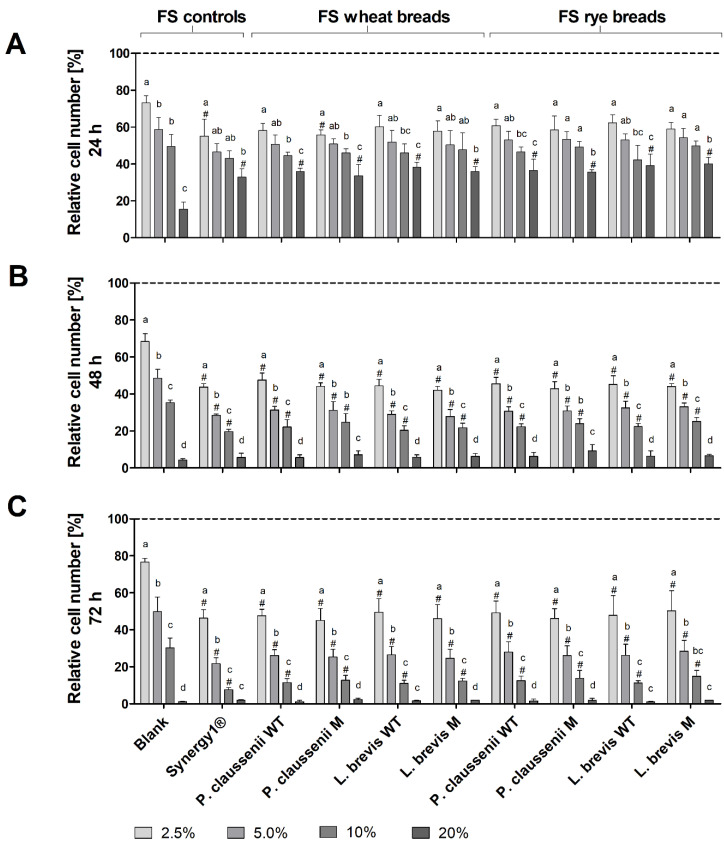
Relative number [%] of LT97 colon adenoma cells after treatment with different concentrations (2.5%, 5%, 10% and 20%) of fermentation supernatants (FS) obtained from controls (blank, Synergy1^®^) and wheat and rye breads for 24 h (**A**), 48 h (**B**) and 72 h (**C**). Breads were generated using wild-type (WT) and mutant (M) strains of *Pediococcus claussenii* (WT: TMW 2.340, M: TMW 2.2123) and *Levilactobacillus brevis* (WT: TMW 1.2112, M: TMW 1.2320). Results were calculated on the basis of the medium control (dashed line), which was set at 100% (mean + SD, *n* = 3). Significant differences between cells treated with FS blank and cells treated with FS of all other samples (^#^ *p* < 0.05) and significant differences between cells treated with different concentrations of FS (^a–d^ *p* < 0.05, different letters represent significantly different results) were obtained by one-way ANOVA and F-test according to Ryan–Einot–Gabriel–Welsh. TMW: Technische Mikrobiologie Weihenstephan.

Treatment of LT97 cells with increasing concentrations of FS obtained from LAB-fermented wheat and rye sourdoughs (Figure 3) as well as the respective breads (Figure 4) resulted in a dose- and time-dependent inhibition of cell growth. Treatment of LT97 cells with 2.5% to 20% FS of wheat and rye sourdoughs for 24 h resulted in average relative cell numbers of 61.4 ± 8.1% to 31.5 ± 6.4% (Figure 3A). A similar reduction was observed after treatment with 2.5% to 20% FS of the fermentation positive control Synergy1^®^ (58.5 ± 8.1% to 21.5 ± 2.7%), while the blank FS reduced cell numbers down to 79.7 ± 7.0% and 7.0 ± 3.8%. The most pronounced effects on cell growth were observed after treatment for 48 h and 72 h (Figure 3B,C). Here, treatment with 2.5% and 5% FS (48 h) or 2.5% and 10% FS (72 h) of LAB fermented wheat and rye sourdoughs as well as with the FS of Synergy1^®^ resulted in significantly lower cell numbers than the treatment with the respective blank FS. In general, cell numbers after incubation with 2.5% to 20% FS blank ranged between 70.7 ± 3.9% and 2.0 ± 1.3% (48 h) as well as 71.1 ± 14.0% and 0.7 ± 0.4% (72 h), while 2.5% to 20% FS of Synergy1^®^ reduced cell numbers to 36.0 ± 7.3% and 3.4 ± 1.6% (48 h) as well as to 38.8 ± 3.5% and 1.1 ± 0.5% (72 h). Similar cell numbers were calculated after treatment with 2.5% to 20% FS of wheat and rye sourdoughs for 48 h (46.5 ± 3.7% and 4.4 ± 2.1%, on average) and 72 h (43.2 ± 10.9% and 1.0 ± 0.4%, on average). Treatment with FS of wheat and rye breads obtained after fermentation with the different LAB strains resulted in a comparable time- and dose-dependent reduction of LT97 cell growth (Figure 4). Again, the most pronounced growth inhibitory effects were observed after treatment of cells for 48 h and 72 h (Figure 4B,C). Here, incubation with the 2.5%, 5%, and 10% FS of Synergy1^®^ and wheat and rye breads led to significantly lower cell numbers than the respective blank FS.

Cell numbers after treatment with the different FS (2.5% to 20%) were as follows: FS blank (24 h: 73.1 ± 3.8 to 15.4± 4.0%, 48 h: 68.4 ± 4.2% to 4.2 ± 0.8%, 72 h: 76.6 ± 2.0 to 1.1 ± 0.2%), FS Synergy1^®^ (24 h: 55.1 ± 9.3% to 32.9 ± 4.4%, 48 h: 43.6 ± 2.0% to 5.5 ± 2.4%, 72 h: 46.4 ± 4.5% to 2.0 ± 0.2%), FS wheat and rye breads (24 h: 59.1 ± 4.5% to 36.8 ± 4.1%, 48 h: 44.4 ± 3.2% to 6.6 ± 2.1%, 72 h: 47.7 ± 6.6% to 1.6 ± 0.7%, on average). In general, no differences in growth inhibitory effects were observed between FS of wheat or rye sourdoughs and breads obtained after fermentation with different wild-type or mutant LAB strains. Furthermore, results from the MTT assay confirm the results obtained from the DAPI assay, indicating that the viability of the LT97 cells was reduced in a nearly time and dose-dependent manner after treatment with FS of sourdoughs and breads (Supplemental Figure S1). However, here, the higher concentrations of 10% and 20% FS showed significant effects on cell viability, while the viability was not reduced after treatment of cells with 2.5% and 5% FS. The latter FS concentrations, which were also calculated as sub-toxic (≤EC_50_) concentrations via nonlinear regression analysis from the DAPI assay results, were used for further experiments.

### 3.6. Genotoxic and Antigenotoxic Effects of FS

**Figure 5:**
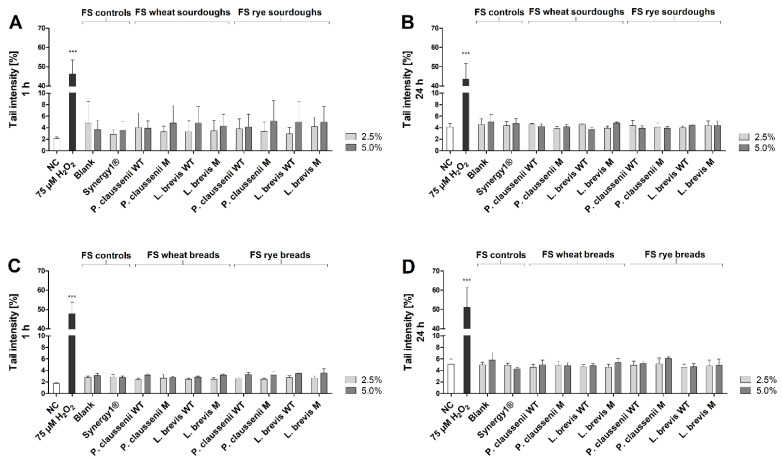
Tail intensity [%] after treatment of LT97 cells with 2.5% and 5% fermentation supernatants (FS) obtained from controls (blank, Synergy1^®^) and wheat and rye sourdoughs for 1 h (**A**) and 24 h (**B**) and with FS obtained from controls (blank, Synergy1^®^) and wheat and rye breads for 1 h (**C**) and 24 h (**D**) (mean + SD, *n* = 3). Sourdoughs and breads were generated using wild-type (WT) and mutant (M) strains of *Pediococcus claussenii* (WT: TMW 2.340, M: TMW 2.2123) and *Levilactobacillus brevis* (WT: TMW 1.2112, M: TMW 1.2320). Significant differences between cells treated with FS and negative controls (NC, PBS (1 h)/medium (24 h)) were checked using the one-way ANOVA. Significant differences between NC and positive control (75 µM H_2_O_2_) were obtained by unpaired Student’s *t*-test (*** *p* < 0.001). TMW: Technische Mikrobiologie Weihenstephan.

After treatment of LT97 cells with FS obtained from the different mutant and wild-type LAB produced wheat and rye sourdoughs and breads for 1 h or 24 h, the measured tail intensities were in a range of 2.4 ± 0.3% and 6.1 ± 0.3% (Figure 5A–D). These values were not significantly different from the tail intensities measured after treatment with the negative controls, which were in the range of 1.8 ± 0.1% and 5.1 ± 0.9%. In contrast, significantly higher tail intensities in the range of 43.7 ± 7.9% and 51.0 ± 10.3% were determined after treatment with the positive control (75 µM H_2_O_2_).

To investigate antigenotoxic effects, cells were challenged with 75 µM H_2_O_2_ for 15 min and pre- or co-incubated with FS obtained from wheat and rye sourdoughs and breads for 1 h or 24 h, respectively. Here, tail intensities were in the range of 24.2 ± 3.3% and 42.6 ± 4.2%. Similar tail intensities were measured after treatment with the positive controls (Supplemental Figure S2).

In general, no differences were observed between the treatments with FS obtained from wild-type or mutant-generated sourdoughs and breads or between wheat and rye sourdoughs and breads.

### 3.7. Induction of Caspase Activity by FS

**Figure 6:**
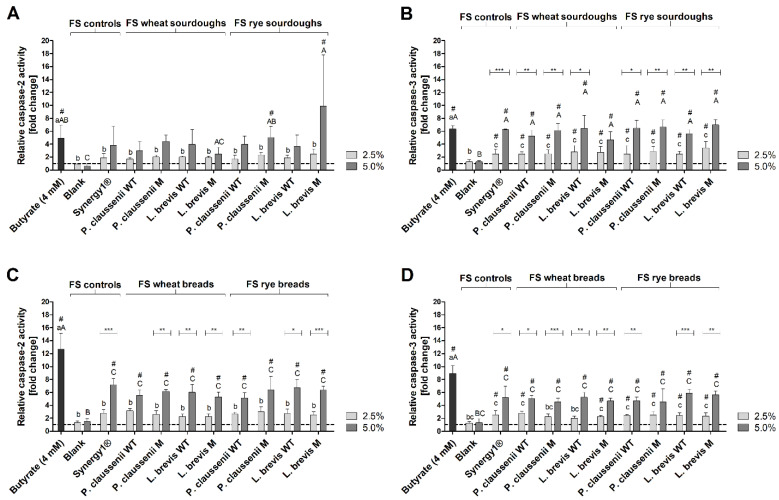
Relative activities of caspase-2 and caspase-3 in LT97 cells after treatment with 2.5% and 5% fermentation supernatant (FS) obtained from controls (blank, Synergy1^®^) and wheat and rye sourdoughs (**A**,**B**) and with FS obtained from controls (blank, Synergy1^®^) and wheat and rye breads (**C**,**D**) as well as 4 mM butyrate for 24 h (mean + SD, *n* = 3). Results are presented as fold changes based on a medium control, which was set 1 (dashed line). Sourdoughs and breads were generated using wild-type (WT) and mutant (M) strains of *Pediococcus claussenii* (WT: TMW 2.340, M: TMW 2.2123) and *Levilactobacillus brevis* (WT: TMW 1.2112, M: TMW 1.2320). Significant differences to the medium control (^#^ *p* < 0.05) and significant differences between cells treated with 2.5% FS as well as butyrate (^a–c^ *p* < 0.05) and 5% FS as well as butyrate (^A–C^ *p* < 0.05) were obtained by two-way ANOVA and F-test according to Ryan–Einot–Gabriel–Welsh. Different letters represent significantly different results. Significant differences between cells treated with 2.5% and 5% FS were obtained by unpaired Student’s *t*-test (* *p* < 0.05, ** *p* < 0.01, *** *p* < 0.001). TMW: Technische Mikrobiologie Weihenstephan.

To examine the pro-apoptotic effects of FS obtained from LAB-fermented wheat and rye sourdoughs as well as breads, the activities of the initiator caspase-2 and the effector caspase-3 were determined as markers for apoptosis. Butyrate, which was used as the positive control, significantly induced caspase-2 and -3 activities in LT97 cells after 24 h of treatment (4.9–12.7-fold) in all experiments (Figure 6A–D).

Furthermore, treatment of LT97 cells with 2.5% and 5% FS of wheat and rye sourdoughs and breads as well as with the FS of Synergy1^®^ also led to a mainly dose-dependent increase in caspase-2 and -3 activities. In general, 5% FS of the samples induced significantly higher activities of these two caspases than the medium control, which was set to 1, and the respective fermentation negative controls (FS blank), which ranged between 0.6-fold and 1.5-fold. FS obtained from wheat and rye sourdoughs insignificantly increased caspase-2 activity in LT97 cells in a range of 1.7–2.5-fold (2.5% FS) and 2.5–9.9-fold (5% FS) (Figure 6A), while caspase-3 was significantly increased after treatment with 2.5% FS (2.5–3.4-fold) and 5% FS (4.7–7.0-fold) (Figure 6B). Similar activities of caspase-2 (FS 2.5%: 1.9-fold, FS 5%: 3.8-fold) and caspase-3 (FS 2.5%: 2.5-fold, FS 5%: 6.3-fold) were determined after treatment with the FS of the fermentation control Synergy1^®^ (Figure 6A,B).

The FS obtained from wheat and rye breads increased caspase-2 activity in the range of 2.3–3.2-fold (2.5% FS) and 5.1–6.7-fold (5% FS) (Figure 6C) as well as caspase-3 activity in the range of 2.0–2.8-fold (2.5% FS) and 4.5–5.9-fold (5% FS) (Figure 6D). Again, treatment with FS of Synergy1^®^ led to similar increases of caspase-2 (2.5% FS: 2.8-fold, 5% FS: 7.2-fold) and caspase-3 (2.5% FS: 2.5-fold, 5% FS: 5.2-fold) (Figure 6C,D).

Treatment of LT97 cells with the FS (5%) of the different sourdough and bread samples for 48 h also resulted in an increase in caspase-2 and -3 activities, but to a lesser extent than the 24 h treatment (Supplemental Figure S3). In general, no differences in the induction of the two caspases were observed between FS of wheat and rye sourdoughs and breads obtained from fermentation with the different LAB wild-type or mutant strains.

### 3.8. Effects of FS on the mRNA Expression of Cell Cycle- and Detoxification-Relevant Target Genes

**Figure 7:**
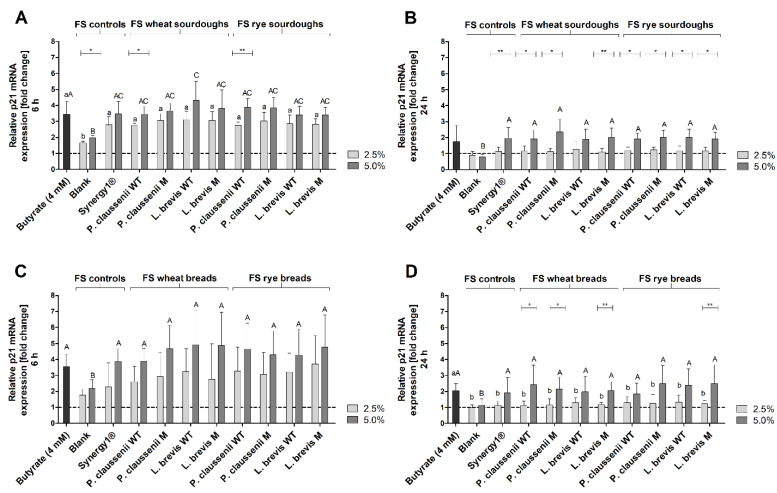
Relative *p21* mRNA expression in LT97 cells after treatment with 2.5% and 5% fermentation supernatants (FS) obtained from controls (blank, Synergy1^®^) and wheat and rye sourdoughs as well as 4 mM butyrate for 6 h (**A**) and 24 h (**B**) and with FS obtained from controls (blank, Synergy1^®^) and wheat and rye breads as well as 4 mM butyrate for 6 h (**C**) and 24 h (**D**) (mean + SD, *n* = 4). Results are presented as fold changes based on a medium control, which was set 1 (dashed line). Sourdoughs and breads were generated using wild-type (WT) and mutant (M) strains of *Pediococcus claussenii* (WT: TMW 2.340, M: TMW 2.2123) and *Levilactobacillus brevis* (WT: TMW 1.2112, M: TMW 1.2320). Significant differences between cells treated with 2.5% FS as well as butyrate (^a,b^ *p* < 0.05) and 5% FS as well as butyrate (^A–C^ *p* < 0.05) were obtained by two-way ANOVA and F-test according to Ryan–Einot–Gabriel–Welsh from log-transformed data. Different letters represent significantly different results. Significant differences between cells treated with 2.5% and 5% FS were obtained by unpaired Student’s *t*-test (* *p* < 0.05, ** *p* < 0.01). TMW: Technische Mikrobiologie Weihenstephan.

After 6 h, *p21* mRNA expression was significantly higher in LT97 cells treated with FS obtained from sourdoughs (2.5% FS: 2.9 ± 0.4-fold, 5% FS: 3.7 ± 0.7-fold, on average) and breads (2.5% FS: 3.1 ± 1.4-fold, 5% FS: 4.5 ± 1.5-fold, on average) than in cells treated with FS obtained from the blank controls (sourdough fermentation series: 2.5% FS: 1.6 ± 0.1-fold, 5% FS: 2.0 ± 0.1-fold, bread fermentation series: 2.5% FS: 1.8 ± 0.4-fold, 5% FS: 2.2 ± 0.5-fold) (Figure 7A,C). Treatment of cells with FS obtained from Synergy1^®^ (sourdough fermentation series: 2.5% FS: 2.8 ± 0.5-fold, 5% FS: 3.5 ± 0.8-fold, bread fermentation series: 2.5% FS: 2.3 ± 1.5-fold, 5% FS: 3.9 ± 0.8-fold) and with the positive control butyrate (sourdough fermentation series: 3.4 ± 0.8-fold, bread fermentation series: 3.5 ± 0.8-fold) resulted in *p21* mRNA levels which were comparable to that induced by FS of sourdoughs and breads (Figure 7A,C).

Compared to 6 h of treatment, *p21* mRNA levels were lower after the incubation of LT97 cells with FS obtained from sourdoughs (2.5% FS: 1.2 ± 0.3-fold, 5% FS: 2.0 ± 0.5-fold, on average) and breads (2.5% FS: 1.2 ± 0.3-fold, 5% FS: 2.2 ± 0.9-fold, on average) for 24 h (Figure 7B,D). Nevertheless, treatment of cells with 5% of the FS resulted in significantly higher *p21* mRNA expression levels than treatment with FS obtained from the respective blank controls (sourdough fermentation series: 2.5% FS: 0.9 ± 0.2-fold, 5% FS: 0.8 ± 0.2-fold, bread fermentation series: 2.5% FS: 1.0 ± 0.2-fold, 5% FS: 1.1 ± 0.4-fold). Comparable levels of *p21* mRNA expression were determined after treatment of cells with the FS of Synergy1^®^ (sourdough fermentation series: 2.5% FS: 1.1 ± 0.3-fold, 5% FS: 2.0 ± 0.7-fold, bread fermentation series: 2.5% FS: 1.1 ± 0.3-fold, 5% FS: 1.9 ± 0.9-fold) and butyrate (sourdough fermentation series: 1.7 ± 1.0-fold, bread fermentation series: 2.1 ± 0.4-fold).

**Figure 8:**
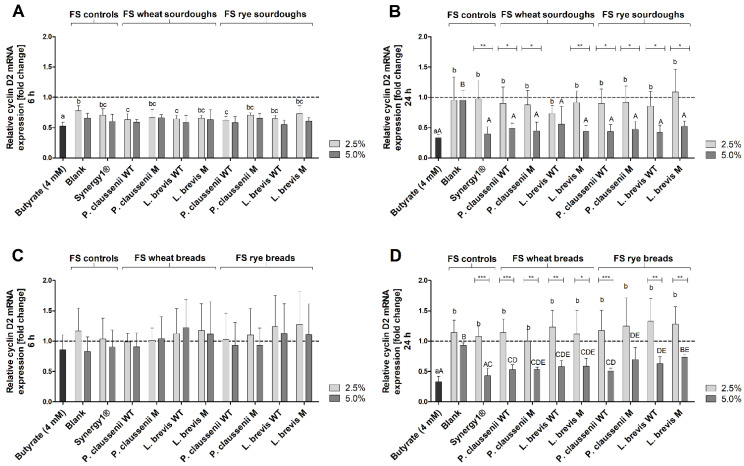
Relative *cyclin D2* mRNA expression in LT97 cells after treatment with 2.5% and 5% fermentation supernatants (FS) obtained from controls (blank, Synergy1^®^) and wheat and rye sourdoughs, as well as 4 mM butyrate for 6 h (**A**) and 24 h (**B**) and with FS obtained from controls (blank, Synergy1^®^) and wheat and rye breads as well as 4 mM butyrate for 6 h (**C**) and 24 h (**D**) (mean + SD, *n* = 4). Results are presented as fold changes based on a medium control, which was set 1 (dashed line). Sourdoughs and breads were generated using wild-type (WT) and mutant (M) strains of *Pediococcus claussenii* (WT: TMW 2.340, M: TMW 2.2123) and *Levilactobacillus brevis* (WT: TMW 1.2112, M: TMW 1.2320). Significant differences between cells treated with 2.5% FS as well as butyrate (^a–c^ *p* < 0.05) and 5% FS as well as butyrate (^A–E^ *p* < 0.05) were obtained by two-way ANOVA and F-test according to Ryan–Einot–Gabriel–Welsh from log-transformed data. Different letters represent significantly different results. Significant differences between cells treated with 2.5% and 5% FS were obtained by unpaired Student’s *t*-test (* *p* < 0.05, ** *p* < 0.01, *** *p* < 0.001). TMW: Technische Mikrobiologie Weihenstephan.

Treatment of LT97 cells with 2.5% and 5% FS obtained from sourdoughs for 6 h resulted in a reduction of *cyclin D2* mRNA levels (2.5% FS: 0.7 ± 0.1-fold, 5% FS: 0.6 ± 0.1-fold, on average) (Figure 8A). Here, the reduction was partly significantly stronger than that of the reduction induced by the FS of the blank control (2.5% FS: 0.8 ± 0.1-fold, 5% FS: 0.7 ± 0.1-fold). After 24 h, 5% of the sourdough FS (2.5% FS: 0.9 ± 0.2-fold, 5% FS: 0.5 ± 0.1-fold, on average) induced a significant reduction of *cyclin D2* mRNA expression compared to the blank FS (2.5% FS: 1.0 ± 0.4-fold, 5% FS: 1.0 ± 0.2-fold) (Figure 8B). Overall, the reduction of *cyclin D2* mRNA levels by the sourdough FS was comparable to the reduction induced by the FS obtained from Synergy1^®^ (6 h incubation: 2.5% FS: 0.7 ± 0.1-fold, 5% FS: 0.6 ± 0.1-fold, 24 h incubation: 2.5% FS: 1.0 ± 0.3-fold, 5% FS: 0.4 ± 0.1-fold), whereas treatment with butyrate resulted in the lowest levels of *cyclin D2* expression (6 h incubation: 0.5 ± 0.1-fold, 24 h incubation: 0.3 ± 0.1-fold) (Figure 8A,B).

The FS obtained from breads and controls (blank, Synergy1^®^) as well as butyrate induced no modulation of *cyclin D2* mRNA levels after 6 h treatment (Figure 8C). In contrast, after 24 h *cyclin D2* mRNA levels were significantly lower (0.6 ± 0.1-fold, on average) after incubation with 5% FS of the breads than after treatment with the respective blank FS (0.9 ± 0.0-fold), whereas treatment with 2.5% of the bread FS (1.2 ± 0.3-fold, on average) or blank FS (1.1 ± 0.2-fold) had no effect on *cyclin D2* mRNA expression. The strongest reduction of *cyclin D2* expression was observed after treatment with butyrate (0.3 ± 0.1-fold) (Figure 8D).

The mRNA expression of *catalase* was not modulated after treatment with FS obtained from sourdoughs and breads for 6 h (Supplemental Figure S4A,C). In contrast, an insignificant induction of *catalase* mRNA expression was observed after the treatment of cells for 24 h with FS of sourdoughs and breads, which was on a similar level than after the incubation of cells with the blank FS, the FS of Synergy1^®^ and butyrate (Supplemental Figure S4 B,D).

*SOD2* mRNA levels were partly significantly higher after 6 h treatment of cells with FS of sourdoughs and breads than after incubation with the blank FS (Supplemental Figure S5A,C). Here, the FS obtained from sourdoughs and breads had similar effects on *SOD2* mRNA levels as the FS of Synergy1^®^ or butyrate. Treatment of LT97 cells with FS of sourdoughs and breads for 24 h had no substantial effects on *SOD2* mRNA expression (Supplemental Figure S5B,D).

In general, no differences in the modulation of *p21* and *cyclin D2* or *catalase* and *SOD2* mRNA levels were observed between the treatments with the FS obtained from the different sourdoughs and breads generated from the wild-type or mutant LAB strains or wheat or rye flour.

### 3.9. Effects of FS on the Expression of Cell Cycle- and Detoxification-Relevant Proteins

**Figure 9:**
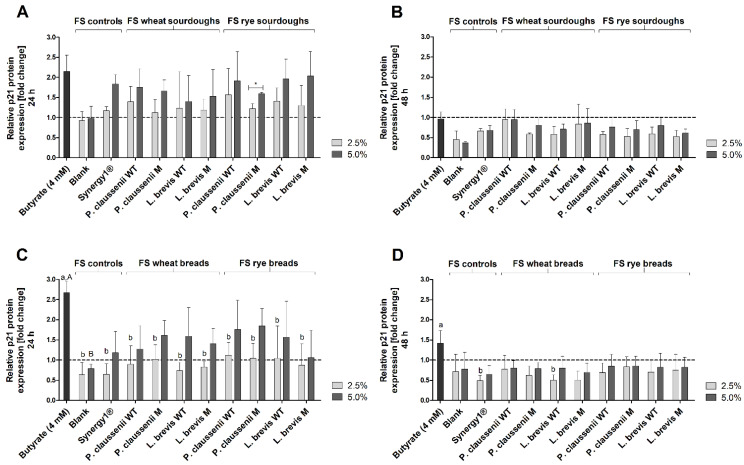
Relative p21 protein expression in LT97 cells after treatment with 2.5% and 5% fermentation supernatants (FS) obtained from controls (blank, Synergy1^®^) and wheat and rye sourdoughs, as well as 4 mM butyrate for 24 h (**A**) and 48 h (**B**) and with FS obtained from controls (blank, Synergy1^®^) and wheat and rye breads as well as 4 mM butyrate for 24 h (**C**) and 48 h (**D**) (mean + SD, *n* = 3). Results are presented as fold changes based on a medium control, which was set 1 (dashed line). Sourdoughs and breads were generated using wild-type (WT) and mutant (M) strains of *Pediococcus claussenii* (WT: TMW 2.340, M: TMW 2.2123) and *Levilactobacillus brevis* (WT: TMW 1.2112, M: TMW 1.2320). Significant differences between cells treated with 2.5% FS as well as butyrate (^a,b^ *p* < 0.05) and 5% FS as well as butyrate (^A,B^ *p* < 0.05) were obtained by one-way ANOVA and F-test according to Ryan–Einot–Gabriel–Welsh. Different letters represent significantly different results. Significant differences between cells treated with 2.5% and 5% FS were obtained by unpaired Student’s *t*-test (* *p* < 0.05). TMW: Technische Mikrobiologie Weihenstephan.

The impact of 2.5% and 5% FS obtained from wheat and rye sourdoughs and breads on the expression of the cell cycle-relevant proteins p21 (Figure 9A–D) and cyclin D2 (Figure 9A–D) were analyzed using Western blot analyses after treatment of LT97 cells.

After 24 h, the relative protein expression of p21 was increased 2.2 ± 0.4-fold by the positive control butyrate (Figure 9A). Treatment of cells with the blank FS caused no change of p21 expression (2.5% FS: 0.9 ± 0.2, 5% FS: 1.0 ± 0.3). In comparison, with a fold-change of 1.8 ± 0.2, treatment with 5% FS Synergy1^®^ resulted in a higher p21 protein expression. A similar induction of p21 expression was observed after incubation of LT97 cells with 5% FS of wheat and rye sourdoughs (1.7 ± 0.5-fold, on average). Due to relatively high standard deviations, this increase was not significantly higher than the p21 expression levels observed for the treatment with the blank FS or 2.5% FS of wheat and rye sourdoughs, which induced a lower expression (1.3 ± 0.4-fold, on average). After 48 h treatment, no induction of p21 protein expression was observed (Figure 9B). Instead, the majority of FS caused a reduction of p21 expression (5% FS blank: 0.5 ± 0.2-fold, 5% FS Synergy1^®^: 0.4 ± 0.0-fold, 5% FS of wheat and rye sourdoughs: 0.8 ± 0.2-fold, on average). Similar results were obtained for the bread fermentation series (Figure 9C,D). Here, butyrate led to a 2.7 ± 0.3-fold increase in p21 protein levels. In contrast, 2.5% and 5% of the blank FS (0.6 ± 0.3-fold, 0.8 ± 0.1-fold) and 2.5% of the FS obtained from Synergy1^®^ (0.7 ± 0.3-fold) and from wheat and rye breads (0.9 ± 0.4-fold, on average) did not enhance the p21 protein expression (Figure 9C). In contrast, 5% FS of Synergy1^®^ (1.2 ± 0.5-fold) and 5% FS of wheat and rye breads (1.5 ± 0.6-fold) tended to increase p21 expression. Again, after 48 h of treatment, no induction of p21 protein expression by the FS of the bread samples or controls (blank, Synergy1^®^) was observed (Figure 9D).

**Figure 10:**
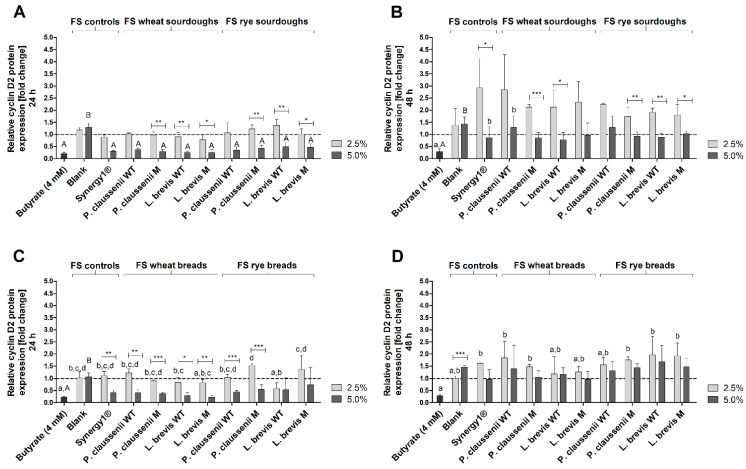
Relative Cyclin D2 protein expression in LT97 cells after treatment with 2.5% and 5% fermentation supernatants (FS) obtained from controls (blank, Synergy1^®^) and wheat and rye sourdoughs, as well as 4 mM butyrate for 24 h (**A**) and 48 h (**B**) and with FS obtained from controls (blank, Synergy1^®^) and wheat and rye breads as well as 4 mM butyrate for 24 h (**C**) and 48 h (**D**) (mean + SD, *n* = 3). Results are presented as fold changes based on a medium control, which was set 1 (dashed line). Sourdoughs and breads were generated using wild-type (WT) and mutant (M) strains of *Pediococcus claussenii* (WT: TMW 2.340, M: TMW 2.2123) and *Levilactobacillus brevis* (WT: TMW 1.2112, M: TMW 1.2320). Significant differences between cells treated with 2.5% FS as well as butyrate (^a–d^ *p* < 0.05) and 5% FS as well as butyrate (^A,B^ *p* < 0.05) were obtained by one-way ANOVA and F-test according to Ryan–Einot–Gabriel–Welsh. Different letters represent significantly different results. Significant differences between cells treated with 2.5% and 5% FS were obtained by unpaired Student’s *t*-test (* *p* < 0.05, ** *p* < 0.01, *** *p* < 0.001). TMW: Technische Mikrobiologie Weihenstephan.

The relative protein expression of cyclin D2 was markedly reduced in LT97 cells after 24 h treatment with butyrate (sourdough fermentation series: 0.2 ± 0.1-fold, bread fermentation series: 0.2 ± 0.0-fold) and 5% FS obtained from Synergy1^®^ (sourdough fermentation series: 0.3 ± 0.0-fold, bread fermentation series: 0.4 ± 0.1-fold) as well as FS obtained from wheat and rye sourdoughs (0.4 ± 0.1-fold, on average) and breads (0.4 ± 0.3-fold, on average) (Figure 10A,C). In most cases, these cyclin D2 protein levels were significantly lower than after treatment with 2.5% and 5% of the blank FS (sourdough fermentation series: 2.5% FS: 1.2 ± 0.1-fold, 5% FS: 1.3 ± 0.2-fold, bread fermentation series: 2.5% FS: 1.0 ± 0.2-fold, 5% FS: 1.1 ± 0.2-fold) or with 2.5% FS of the sourdough (1.0 ± 0.3-fold, on average) and bread samples (1.0 ± 0.4-fold, on average). After 48 h treatment, protein levels of cyclin D2 were only reduced by butyrate (sourdough fermentation series: 0.3 ± 0.3-fold, bread fermentation series: 0.3 ± 0.1-fold), while all other samples did not change cyclin D2 levels or led to an insignificant increase, as observed after the treatment of cells with 2.5% FS of Synergy1^®^ (sourdough fermentation series: 2.9 ± 1.2-fold, bread fermentation series: 1.6 ± 0.0-fold) and FS obtained from sourdough (2.1 ± 0.7-fold, on average) as well as FS obtained from bread (1.6 ± 0.5-fold, on average) samples (Figure 10B,D).

In general, no significant differences in the induction of p21 and the reduction of cyclin D2 protein levels were observed between the treatments with the FS obtained from the different sourdoughs and breads generated from the mutant or wild-type LAB strains or from wheat or rye flour.

In addition, protein expression of catalase (Supplemental Figure S6) and SOD2 (Supplemental Figure S7) was analyzed in LT97 cells after treatment with 2.5% and 5% FS of sourdough and bread samples. Here, the different FS did not change, or induced only a marginal increase in SOD2 and catalase protein levels. No differences in SOD2 and catalase protein levels were observed between the treatments with the blank FS, the FS of Synergy1^®^, and FS obtained from the wheat and rye sourdough and bread samples generated from LAB mutant or wild-type strains. Representative images of Western blots are presented in Supplemental Figures S8 and S9.

## 4. Discussion

Sourdough fermentation has a long tradition in the production of breads, and several studies indicate that the use of LAB as starter cultures and the resulting fermentation process offers the possibility to produce breads with beneficial effects on human health [49]. These positive health effects may result from the production of EPS, which is formed by LAB during the fermentation process [6,8,49,50,51]. Furthermore, the use of special β-glucan-producing LAB as starters in sourdough fermentation offers the possibility to naturally enrich breads and baked goods with β-glucan, to improve sensory and rheological properties and generate foods with an additional health-promoting value [12,13]. For β-glucan, in general, several health-related effects have been described already [14,52,53]. Usually, HoPS are formed in relatively high yields of up to 40 g/L using sucrose as substrate. In contrast, the intracellular synthesis of HePS and β-glucan results in lower yields of less than 1 g/L [9,54]. Nevertheless, the advantage of using β-glucan-producing LAB as starter for sourdough fermentation is that already low amounts of β-glucan sufficiently increase the viscosity of the doughs and exert structure-forming effects because of their network-like capsular structure [12,13].

In the present study, the fermentation of wheat and rye sourdoughs resulted in β-glucan concentrations of 119.9–519.4 µg/g in sourdoughs and 100.2–674.2 µg/g in breads. These results are in line with results obtained in other studies. For example, similar concentrations of up to 624.7 µg/g β-glucan were found in wheat and rye sourdoughs after fermentation with *L. brevis* and *P. claussenii* using different fermentation conditions [12]. Pérez-Ramos et al. [55] investigated the production of *O2*-substituted (1,3)-β-D-glucan in different flours fermented with *Pediococcus parvulus* 2.6 and determined the highest values of up to 659.4 mg/L β-glucan after fermentation of oat flour for 64 h. In the present study, β-glucan was formed by the wild-type strains of *L. brevis* and *P. claussenii*, whereas the mutant strains were not capable of glucan production. The low amounts of β-glucan detected in fermented wheat and rye sourdoughs and breads using the mutant strains might result from non-specific cross-reactions with, for example, yeast β-glucan or other polysaccharides, as discussed also by Bockwoldt et al. [12]. Interestingly, the moisture content of wild-type LAB-generated rye breads was 2-fold higher (46%, on average, data not shown) than that of rye breads generated with mutant LAB strains (23%, on average, data not shown). These results indicate a higher water holding capacity, at least for the β-glucan-enriched rye breads, whereas in wheat breads generated with wild-type and mutant strains, similar water contents were measured. Furthermore, a similar result was observed for another EPS of LAB, the high-molecular dextran, which is able to bind high amounts of water [9].

No distinct differences were observed between sourdoughs or breads generated with mutant or wild-type strains of *L. brevis* and *P. claussenii* regarding the composition of the main nutrients, though the sourdoughs and breads fermented with the wild-type strain of *P. claussenii* contained more dietary fiber (up to 7.5%) than the sourdoughs and breads generated with the mutant strain of *P. claussenii*. Furthermore, breads made of *P. claussenii* fermented sourdoughs contained higher amounts of total dietary fiber than the respective sourdoughs. These differences might result from the redistribution of dietary fiber and the formation of resistant starch during the bread-making process, as also observed and discussed by others [50,56,57,58,59,60]. In addition, the fermentation conditions, for example, the strains used as starters, might affect the dietary fiber and resistant starch contents [56,61]. In general, contents of fiber, protein and ash in wheat and rye sourdoughs and breads are similar to that observed in different other studies [57,59,60,61,62,63], whereby occurring differences between our results and the nutrient composition observed by others, particularly dietary fiber, might be explained by the raw material used for fermentation, such as the type of flours as well as the fermentation conditions or detection methods.

Next, the fermentation profile of the wheat and rye sourdoughs and breads generated with wild-type and mutant strains of *L. brevis* and *P. claussenii* was investigated after in vitro simulated digestion and batch fermentation. Compared to the blank FS, the fermentation of sourdoughs and breads resulted in a reduction in pH values and the formation of SCFAs with a slight shift of molar ratios towards propionate and a more intense shift towards butyrate formation. This shift was comparable to that observed for the FS of the positive control Synergy1^®^. Similar results were obtained already in other studies investigating the fermentation profile of dietary fiber-rich foods, such as oat [64], barley [20], nuts [65], wheat aleurone [66] or bread samples [67]. The formation of SCFA, together with an increase in molar ratios of butyrate [68,69] and also propionate [42,70,71], might be associated with chemopreventive effects. In the present study, no or only marginal differences in the fermentation profile were observed between the different sourdough or bread samples or between samples generated with wild-type or mutant LAB strains. These results indicate that, during fermentation, the high complexity of the food matrix masks the effects of the relatively low amounts of β-glucan produced by the wild-type strains of the LAB. In contrast, in a recent study, the fermentation profile of isolated β-glucan and cell preparations obtained from *L. brevis* and *P. claussenii* revealed clear differences between wild-type and mutant strains [26]. For example, higher concentrations of SCFA were measured in FS obtained from wild-type strains than in FS of mutant strains with a more pronounced shift of molar ratios of SCFA towards propionate instead of butyrate; and concentrations of ammonia were lower in FS obtained from wild-type LAB strains than in FS of the mutant strains. However, ammonia levels, in general, were significantly higher than in the FS of the blank control due to the low purity of the samples and high residual protein levels. Therefore, the investigation of in situ-enriched LAB β-glucan in a complex food matrix is mandatory to avoid such artifacts. Nevertheless, the achieved amounts of β-glucan were not sufficient to generate differences in the fermentation profiles between samples obtained from wild-type and mutant strains.

Furthermore, proteins present in sourdoughs and breads contribute to proteolytic fermentation, resulting in comparable ammonia concentrations as measured in the FS of the blank control. High concentrations of ammonia are associated with tumor-promoting potential, whereas fermentable carbohydrates reduce ammonia concentrations and may contribute to colon cancer prevention [72,73]. An insignificant reduction of ammonia was observed in the FS of rye sourdoughs, for example. In contrast, the fermentation of pure dietary fiber, such as Synergy1^®^, resulted in a significant and clear reduction of ammonia. A significant reduction of ammonia levels was also observed in FS of β-glucans from different species, such as oat, barley and yeast, as well as curdlan compared to the respective blank FS [26]. A reduction of ammonia levels in FS of dietary fiber-rich foods, such as different types of breads [67], was also observed earlier.

In the present study, marginally higher values of butyrate were measured in the FS obtained from sourdoughs than in the FS of bread samples. Butyrate is one end product of dietary fiber fermentation. Since the amounts of dietary fiber in sourdoughs and breads were similar, other effects might be responsible for the observed differences in the fermentation profile of sourdoughs and breads, such as a different composition of the feces matrix resulting from different feces donors used for the fermentation of the sourdoughs and breads. As reflected already at time point t0 of fermentation, the two fermentation series differed markedly regarding their bacterial profile. The FPs of the bread fermentation series contained a large number of bacterial families with higher proportions of propionate-producing species (*Bacteroidaceae*, Supplemental Table S1), whereas the bacterial community was less diverse in the sourdough fermentation series containing higher proportions of butyrate-producing species such as *Ruminococcaceae* (Supplemental Table S1) [74]. Nevertheless, the results indicate that the sourdough and bread samples similarly modulate the abundance of bacteria species. Most obviously, proportions of *Bifidobacteriaceae* were increased in FP of sourdoughs and breads compared to the blank FP, independently of the used wild-type or mutant LAB strain or the type of flour used for sourdough fermentation. This specific increase was comparable to that observed in the FPs of Synergy1^®^ and corresponds to the bifidogenic effects described for inulin in the in vitro colonic fermentation models [75]. As reviewed, in vitro and in vivo studies indicate that inulin, β-glucan and dietary fiber in general exert bifidogenic and prebiotic effects [75,76,77,78]. Furthermore, Costabile et al. [79] demonstrated an increase of *Bifidobacteriaceae* in a healthy donor group after the administration of fermented wheat sourdough bread compared to breads generated without sourdough fermentation, using in vitro batch culture experiments. In contrast, in a human intervention study, no differences in stool microbiota composition were observed between the groups consuming whole grain sourdough wheat bread and white wheat bread [80]. However, the results of the present study indicate that the LAB-generated sourdoughs and breads have the potential to establish or maintain a favorable and healthy environment in the colon by the formation of SCFA. The reduction of pH values might be associated with prebiotic effects by stimulating the growth of acidophilic microbial strains, such as *Bifidobacteriaceae*, or protection against the growth of pathogenic strains [81,82,83]. Furthermore, butyrate as a chemopreventive metabolite of dietary fiber fermentation particularly exhibits colon health-promoting and anticancer effects [82,84]. Butyrate has a key role as a so-called “blocking agent” in primary chemoprevention (characterized by enhancing the elimination of ROS and detoxification) and as a so-called “suppressing agent” in secondary chemoprevention of colon cancer (characterized by inhibition of proliferation and induction of apoptosis) [69]. Several studies showed that butyrate inhibits the growth of colon adenoma or carcinoma cells [71,84,85,86,87]. Therefore, butyrate might be mainly responsible for the observed reduction of LT97 cell growth and viability after treatment with FS obtained from sourdoughs and breads. Growth inhibitory effects were also observed for propionate [70,71], so synergistic effects of different SCFA together with other sourdough and bread fermentation metabolites can be expected. Furthermore, the results are in line with the results obtained in other studies that investigated similar growth inhibitory properties of dietary fiber-rich foods, such as aleurone [66], bread [42], nuts [53] or oat- and barley flakes [19,20].

The activities of the initiator caspase-2 and the effector caspase-3 were significantly induced by the FS obtained from sourdoughs and breads, indicating that the observed growth inhibition may be at least partly mediated by apoptotic processes. An increase in caspase-3 activity as one marker of apoptosis was already demonstrated in recent studies investigating the chemopreventive effects of different dietary fiber sources in colon adenoma or carcinoma cells [19,20,66,88]. Caspase-2 can be activated by several intrinsic and extrinsic stimuli and triggers cell cycle arrest and apoptosis. For example, caspase-2 is activated by genotoxic stress and DNA damage, but also due to endoplasmic reticulum stress, the activation of death receptors, growth factor deprivation, metabolic stress or heat shock [89,90,91].

In the present study, tail intensities after treatment of LT97 cells with FS obtained from sourdoughs and breads were not increased. Therefore, genotoxic effects as a possible cause for caspase-2 activation may be excluded. Again, the main effects regarding caspase activation might be due to butyrate, which also significantly increased caspase activities in the present study. Putaala et al. [92], for example, also observed an increase in caspase-2 and -3 activities after treatment of Caco-2 cells with 5 mM butyrate and FS obtained from polydextrose. In general, for butyrate-mediated caspase activation and the induction of apoptosis, intrinsic and extrinsic pathways have been postulated [93].

Furthermore, the induction of *p21* mRNA (6 h, 24 h) and protein expression (24 h), as well as the reduction of *cyclin D2* mRNA (6 h, 24 h) and protein levels (24 h) by FS obtained from sourdoughs and breads, might also be attributed to butyrate. Butyrate regulates the expression of cell cycle-relevant genes, such as *p21* and *cyclins* in colon adenoma and carcinoma cells via its function as a histone deacetylase inhibitor resulting in cell cycle arrest [68,84,86,94]. Similar modulation of *p21* and *cyclin D2* by FS obtained from different dietary fiber-rich sources, such as nuts [53] was detected in LT97. Induction of *p21* was also observed in LT97 and HT29 cells after treatment with FS obtained from wheat aleurone [88]. In addition, treatment of HT29 colon carcinoma cells with FS of wheat aleurone resulted in an increase of cells in the G0/G1 phase [66]. Chai et al. [95] observed an increase in *p21* mRNA in Lim 1215 colorectal cancer cells after treatment with 4 mM butyrate in a time period of 3–12 h, which precedes the induction of DEVD-caspases [96]. This is in line with the high increase in *p21* mRNA observed in the present study after 6 h of treatment with butyrate and FS obtained from sourdoughs and breads and the induction of caspases after 24 h. However, regulation of cell cycle-relevant genes and proteins such as *p21* can also result from the activation of caspases. For example, the induction of caspase-2 results in the probably cell-specific induction of *p21* by stabilization of p53, which is mediated by the cleavage of Mdm2 to maintain genomic stability [89,90]. Therefore, the regulation of cell cycle-relevant genes or proteins, as well as the induction of apoptosis by FS obtained from sourdoughs and breads may result from a complex regulatory system, and the exact mechanisms have to be elucidated in further studies. Nevertheless, the results of the present study indicate a cell cycle-regulating and apoptosis-inducing potential of FS obtained from wheat and rye sourdoughs and breads in colon adenoma cells, which may be at least partly responsible for the observed growth inhibitory effects. These effects might be mainly attributable to butyrate as a chemopreventive metabolite of dietary fiber fermentation. However, it cannot be excluded that other components or metabolites resulting from the fermentation of the complex food matrix by the human fecal microbiota may contribute to the observed effects. Furthermore, FS of sourdoughs and breads seem to exert mainly secondary chemopreventive effects mediated by the induction of cell cycle arrest and apoptosis rather than primary chemopreventive effects, since no or only a small induction of antioxidant relevant enzymes (on the mRNA and protein level) and no significant antigenotoxic potential were observed. In contrast, an increase in mRNA levels of antioxidant- and detoxification-relevant genes, such as *catalase* or *GSTP1*, and antigenotoxic potential were observed in LT97 cells after treatment with FS of different nuts, such as hazelnuts [45] or pistachios [97], indicating that other components or fermentation metabolites of the specific nut food matrix might have induced these effects.

## 5. Conclusions

In conclusion, the results of the present study demonstrate that wheat and rye sourdoughs and breads obtained by fermentation with wild-type and mutant strains of the LAB *L. brevis* and *P. claussenii* lead to comparable fermentation profiles, which may be associated with prebiotic effects. Furthermore, the FS of the different sourdoughs and breads exhibit secondary chemopreventive potential via inhibition of LT97 cell growth, which is mediated by apoptotic and cell cycle regulatory effects. Here, the in situ-formed LAB β-glucan has no additional effect. However, it cannot be excluded that the chemopreventive endpoints examined in the in vitro cell experiments might not be sensitive enough to disclose the small differences in the β-glucan contents between the sourdough and bread samples generated with wild-type and mutant strains of the LAB. Nevertheless, the present study provides important insights into the chemopreventive and prebiotic effects of LAB generated sourdoughs and breads in general.

## Figures and Tables

**Table 1:** Primer Sequences.

Genes	Sequence
*Catalase*	Forward 5′-TGG ACA AGT ACA ATG CTG AG-3′
	Reverse 5′-TTA CAC GGA TGA ACG CTA AG-3′
*SOD2*	Forward 5′-GCC CTG GAA CCT CAC ATC AAC-3′
	Reverse 5′-CAA CGC CTC CTG GTA CTT CTC-3′
*p21*	Forward 5′-CAC TGT CTT GTA CCC TTG TG-3′
	Reverse 5′-CTT CCT CTT GGA GAA GAT CAG-3′
*Cyclin D2*	Forward 5′-CCA CCG ACT TTA AGT TTG CC-3′
	Reverse 5′-CTT TGA GAC AAT CCA CGT CTG-3′
*β-Actin*	Forward 5′-AGA GCC TCG CCT TTG CCG AT-3′
	Reverse 5′-CCC ACG ATG GAG GGG AAG AC-3′
*GAPDH*	Forward 5′-CAA CAG CGA CAC CCA CTC CT-3′
	Reverse 5′-CAC CCT GTT GCT GTA GCC AAA-3′

**Table 2:** Nutrient composition of sourdoughs and breads obtained from fermentations with lactic acid bacteria.

	Wheat					Rye				
	Total Dietary Fiber [%]	Protein [%]	Fat [%]	Ash [%]	β-Glucan [µg/g]	Total Dietary Fiber [%]	Protein [%]	Fat [%]	Ash [%]	β-Glucan [µg/g]
**Sourdough**										
*P. claussenii* WT	17.2	11.3	0.8	0.7	119.9	22.8	9.2	0.9	1.4	169.4
*P. claussenii* M	13.0	11.2	0.8	0.7	35.7	19.1	8.7	1.2	1.3	40.3
*L. brevis* WT	15.9	11.1	0.9	0.7	248.6	20.3	9.8	1.3	1.4	519.4
*L. brevis* M	15.5	10.9	0.8	0.7	25.0	20.0	9.6	1.4	1.4	27.6
**Bread**										
*P. claussenii* WT	20.5	11.8	1.5	2.9	188.3	26.5	9.6	1.5	3.3	674.2
*P. claussenii* M	13.0	11.9	1.5	2.8	0.0	23.2	9.4	1.9	3.4	0.0
*L. brevis* WT	14.3	11.1	1.5	2.9	100.2	19.8	9.6	1.3	3.4	312.2
*L. brevis* M	16.5	11.5	1.4	2.8	30.1	19.3	10.1	1.7	3.4	0.0

Results are presented as means of two independent measurements in freeze-dried samples. *Pediococcus (P.) claussenii* (WT: TMW 2.340, M: TMW 2.2123) and *Levilactobacillus* (L.) *brevis* (WT: TMW 1.2112, M: TMW 1.2320). M: mutant, TMW: Technische Mikrobiologie Weihenstephan, WT: wild-type.

**Table 3:** pH values, SCFA concentrations, molar ratios of main SCFAs and ammonia concentrations in FS of sourdoughs.

FS	pH ^(a)^	SCFA [mmol/L] ^(a)^			∑ SCFAs [mmol/L]	Molar Ratio SCFAs [%]	Ammonia [mmol/L] ^(b)^
		Acetate	Propionate	Butyrate		Acetate:Propionate:Butyrate	
**Controls**							
Blank	6.7	23.1	8.6	6.4	38.0	60.6:22.5:16.9	19.7 ± 1.0 ^a^
Synergy1^®^	5.2	64.2	12.1	25.8	102.1	62.9:11.8:25.3	5.4 ± 0.3 ^b^
**Wheat sourdoughs**							
*P. claussenii* WT	5.4	61.1	13.8	22.4	97.3	62.8:14.2:23.0	14.8 ± 1.1 ^c^
*P. claussenii* M	5.3	62.3	14.5	24.2	100.9	61.7:14.3:24.0	16.9 ± 0.8 ^c,d^
*L. brevis* WT	5.3	65.5	14.8	24.7	104.9	62.4:14.1:23.5	17.4 ± 0.4 ^a,d^
*L. brevis* M	5.4	61.0	13.9	22.2	97.1	62.9:14.3:22.8	19.0 ± 0.2 ^a,d^
**Rye sourdoughs**							
*P. claussenii* WT	5.5	58.3	13.6	21.5	93.4	62.4:14.6:23.0	17.2 ± 0.5 ^c,d^
*P. claussenii* M	5.5	58.2	13.9	22.1	94.2	61.7:14.8:23.5	17.3 ± 1.3 ^c,d^
*L. brevis* WT	5.5	58.2	13.6	21.8	93.6	62.1:14.6:23.3	16.7 ± 0.4 ^c,d^
*L. brevis* M	5.5	54.5	13.5	20.9	88.9	61.3:15.2:23.5	16.2 ± 1.8 ^c,d^

Results are presented as means of two ^(a)^ or as means ± SD of three ^(b)^ independent measurements. Significant differences between ammonia concentrations measured in fermentation supernatants (FS) of blank, Synergy1^®^ and FS obtained from sourdoughs enriched with lactic acid bacteria (LAB) of wild-type (WT) and mutant (M) strains of *P. claussenii* (WT: TMW 2.340, M: TMW 2.2123) and *L. brevis* (WT: TMW 1.2112, M: TMW 1.2320) (^a–d^ *p* < 0.05, different letters represent statistically different results) were obtained by one-way ANOVA and F-test according to Ryan–Einot–Gabriel–Welsh. SCFA: short-chain fatty acid, TMW: Technische Mikrobiologie Weihenstephan.

**Table 4:** pH values, SCFA concentrations, molar ratios of main SCFAs and ammonia concentrations in FS of breads.

FS	pH ^(a)^	SCFA [mmol/L] ^(a)^			∑ SCFAs [mmol/L]	Molar Ratio SCFAs [%]	Ammonia [mmol/L] ^(b)^
		Acetate	Propionate	Butyrate		Acetate:propionate:butyrate	
**Controls**							
Blank	6.5	23.9	8.7	5.5	38.1	62.7:22.8:14.5	13.6 ± 0.9 ^a^
Synergy1^®^	5.0	57.8	15.6	22.2	95.6	60.5:16.3:23.2	2.2 ± 0.3 ^b^
**Wheat breads**							
*P. claussenii* WT	5.2	58.3	17.4	17.5	93.1	62.6:18.6:18.8	15.7 ± 1.4 ^a^
*P. claussenii* M	5.2	59.2	17.4	18.0	94.6	62.6:18.4:19.0	16.4 ± 0.6 ^a^
*L. brevis* WT	5.2	59.5	17.5	17.8	94.8	62.8:18.5:18.7	16.1 ± 0.8 ^a^
*L. brevis* M	5.2	58.3	17.3	17.4	93.1	62.7:18.6:18.7	17.0 ± 1.0 ^a^
**Rye breads**							
*P. claussenii* WT	5.2	56.1	17.0	16.5	89.6	62.6:19.0:18.4	13.9 ± 2.1 ^a^
*P. claussenii* M	5.2	56.6	17.3	16.9	90.8	62.4:19.0:18.6	14.3 ± 1.6 ^a^
*L. brevis* WT	5.2	58.0	17.5	17.1	92.6	62.6:18.9:18.5	13.2 ± 3.6 ^a^
*L. brevis* M	5.2	55.9	17.3	16.7	89.9	62.2:19.2:18.6	13.8 ± 0.1 ^a^

Results are presented as means of two ^(a)^ or as means ± SD of three ^(b)^ independent measurements. Significant differences between ammonia concentrations measured in fermentation supernatants (FS) of blank, Synergy1^®^ and FS obtained from breads resulting from sourdough fermentation with lactic acid bacteria (LAB) of wild-type (WT) and mutant (M) strains of *P. claussenii* (WT: TMW 2.340, M: TMW 2.2123) and *L. brevis* (WT: TMW 1.2112, M: TMW 1.2320) (^a,b^ *p* < 0.05, different letters represent statistically different results) were obtained by one-way ANOVA and F-test according to Ryan–Einot–Gabriel–Welsh. SCFA: short-chain fatty acid, TMW: Technische Mikrobiologie Weihenstephan.

## Data Availability

The data presented in the study are available on request from the corresponding author.

## Supplementary Materials

The following supporting information can be downloaded at: https://www.mdpi.com/article/10.3390/nu14071510/s1, Figure S1. Relative viability [%] of LT97 colon adenoma cells after treatment with different concentrations (2.5%, 5%, 10% and 20%) of fermentation supernatants (FS) obtained from controls (blank, Synergy1^®^) and wheat and rye sourdoughs (A) and FS obtained from controls (blank, Synergy1^®^) and wheat and rye breads (B) for 24 h, 48 h and 72 h.; Figure S2. Tail intensity [%] after treatment of LT97 cells with 2.5% and 5% fermentation supernatants (FS) obtained from controls (blank, Synergy1^®^) and wheat and rye sourdoughs for 1 h (A) and 24 h (B) and with FS obtained from controls (blank, Synergy1^®^) and wheat and rye breads for 1 h (C) and 24 h (D) (mean + SD, n = 3) after challenging with 75 µM H2O2 for 15 min.; Figure S3. Relative activities of caspase-2 and caspase-3 in LT97 cells after treatment with 2.5% and 5% fermentation supernatant (FS) obtained from controls (blank, Synergy1^®^) and wheat and rye sourdoughs (A, B) and with FS obtained from controls (blank, Synergy1^®^) and wheat and rye breads (C, D) as well as 4 mM butyrate for 48 h (mean + SD, n = 3).; Figure S4. Relative catalase mRNA expression in LT97 cells after treatment with 2.5% and 5% fermentation supernatants (FS) obtained from controls (blank, Synergy1^®^) and wheat and rye sourdoughs as well as 4 mM butyrate for 6 h (A) and 24 h (B) and with FS obtained from controls (blank, Synergy1^®^) and wheat and rye breads as well as 4 mM butyrate for 6 h (C) and 24 h (D) (mean + SD, n = 4).; Figure S5. Relative SOD2 mRNA expression in LT97 cells after treatment with 2.5% and 5% fermentation supernatants (FS) obtained from controls (blank, Synergy1^®^) and wheat and rye sourdoughs, as well as 4 mM butyrate for 6 h (A) and 24 h (B) and with FS obtained from controls (blank, Synergy1^®^) and wheat and rye breads as well as 4 mM butyrate for 6 h (C) and 24 h (D) (mean + SD, n = 4).; Figure S6. Relative catalase protein expression in LT97 cells after treatment with 2.5% and 5% fermentation supernatants (FS) obtained from controls (blank, Synergy1^®^) and wheat and rye sourdoughs, as well as 4 mM butyrate for 24 h (A) and 48 h (B) and with FS obtained from controls (blank, Synergy1^®^) and wheat and rye breads as well as 4 mM butyrate for 24 h (C) and 48 h (D) (mean + SD, n = 3).; Figure S7. Relative SOD2 protein expression in LT97 cells after treatment with 2.5% and 5% fermentation supernatants (FS) obtained from controls (blank, Synergy1^®^) and wheat and rye sourdoughs, as well as 4 mM butyrate for 24 h (A) and 48 h (B) and with FS obtained from controls (blank, Synergy1^®^) and wheat and rye breads as well as 4 mM butyrate for 24 h (C) and 48 h (D) (mean + SD, n = 3).; Figure S8. Representative images of Western blot analyses used for protein expression analyses of p21, cyclin D2, catalase (Cat), SOD2 and GAPDH (reference protein) in LT97 cells after treatment with fermentation supernatants (FS) obtained from wheat and rye sourdoughs generated with wild-type (WT) and mutant (M) strains of Pediococcus claussenii (WT: TMW 2.340, M: TMW 2.2123) and Levilactobacillus brevis (WT: TMW 1.2112, M: TMW 1.2320). Supplemental Figure S9. Representative images of Western blot analyses used for protein expression analyses of p21, cyclin D2, catalase (Cat), SOD2 and GAPDH (reference protein) in LT97 cells after treatment with fermentation supernatants (FS) obtained from wheat and rye breads generated with wild-type (WT) and mutant (M) strains of Pediococcus claussenii (WT: TMW 2.340, M: TMW 2.2123) and Levilactobacillus brevis (WT: TMW 1.2112, M: TMW 1.2320). Table S1. Proportion of main bacterial families in FP of wheat and rye sourdoughs and breads.
