# Supplementary material for: Use of the β-Glucan-Producing Lactic Acid Bacteria Strains Levilactobacillus brevis and Pediococcus claussenii for Sourdough Fermentation—Chemical Characterization and Chemopreventive Potential of In Situ-Enriched Wheat and Rye Sourdoughs and Breads
Source: Nutrients. 2022 Apr 5;14(7):1510. doi: 10.3390/nu14071510 (PMC9002695; doi:10.3390/nu14071510)
Supplement: Supplementary file 1 [file nutrients-14-01510-s001.zip › nutrients-1660235-supplementary.pdf]

*Supplementary Material*

# Use of the β-glucan-producing lactic acid bacteria strains *Levilactobacillus brevis* and *Pediococcus claussenii* for sourdough fermentation - Chemical characterization and chemopreventive potential of *in situ*-enriched wheat and rye sourdoughs and breads

Wiebke Schlörmann [1,*], Julia A. Bockwoldt [2], Sabine M. Hübner [1], Elisa Wittwer [1], Sarah Reiners [3,5], Stefan Lorkowski [4,5], Christine Dawczynski [3,5], Matthias A. Ehrmann [2] and Michael Glei [1]

[1] Friedrich Schiller University Jena, Institute of Nutritional Sciences, Department of Applied Nutritional Toxicology, Dornburger Straße 24, 07743 Jena, Germany; wiebke.schloermann@uni-jena (W.S.); huebner.bine@web.de (S.M.H.); elisa.wittwer@uni-jena.de (E.W.); michael.glei@uni-jena.de (M.G.)

[2] Technical University of Munich, Chair of Microbiology, Gregor-Mendel-Straße 4, 85354 Freising, Germany; julia.bockwoldt@tum.de; matthias.ehrmann@tum.de (M.A.E.)

[3] Friedrich Schiller University Jena, Institute of Nutritional Sciences, Junior Research Group Nutritional Concepts, Dornburger Straße 29, 07743 Jena; sarah.reiners@uni-jena.de (S.R.); christine.dawczynski @uni-jena.de (C.D.)

[4] Friedrich Schiller University Jena, Institute of Nutritional Sciences, Department of Nutritional Biochemistry and Physiology, Dornburger Straße 25, 07743 Jena, Germany; Stefan.lorkowski@uni-jena.de (S.L.)

[5] Competence Cluster for Nutrition and Cardiovascular Health (nutriCARD) Halle-Jena-Leipzig, Germany

* Correspondence: wiebke.schloermann@uni-jena.de (W.S.); Tel.: +49 3641 949694

**Supplemental Table S1.** Proportion of main bacterial families in FP of wheat and rye sourdoughs and breads

| | Proportion of bacteria families [%] | | | | |
|---|---|---|---|---|---|
| | **Bacteroidaceae** | **Bifidobacteriaceae** | **Coriobacteriaceae** | **Lachnospiraceae** | **Ruminococcaceae** |
| **Sourdough** | | | | | |
| T0 | 3.0 | 15.5 | 5.3 | 29.8 | 26.0 |
| Blank | 9.3 | 20.5 | 4.6 | 20.4 | 23.6 |
| Synergy1® | 5.7 | 33.2 | 6.5 | 16.8 | 15.4 |
| Wheat, *P. claussenii* WT | 6.6 | 28.4 | 6.8 | 18.4 | 22.4 |
| Wheat, *P. claussenii* M | 6.8 | 30.1 | 6.7 | 16.1 | 19.9 |
| Wheat, *L. brevis* WT | 6.2 | 30.1 | 5.6 | 15.8 | 21.0 |
| Wheat, *L. brevis* M | 6.0 | 27.6 | 6.7 | 17.0 | 21.8 |
| Rye, *P. claussenii* WT | 5.8 | 27.2 | 5.6 | 17.4 | 22.3 |
| Rye, *P. claussenii* M | 5.5 | 28.6 | 6.2 | 17.3 | 23.2 |
| Rye, *L. brevis* WT | 5.3 | 27.9 | 5.8 | 19.5 | 22.6 |
| Rye, *L. brevis* M | 5.9 | 26.3 | 6.5 | 18.7 | 22.6 |
| **Bread** | | | | | |
| T0 | 7.4 | 3.0 | 2.3 | 28.1 | 20.6 |
| Blank | 15.3 | 3.5 | 2.6 | 20.2 | 17.7 |
| Synergy1® | 11.1 | 13.8 | 10.2 | 19.1 | 13.8 |
| Wheat, *P. claussenii* WT | 10.6 | 13.9 | 11.3 | 16.4 | 13.3 |
| Wheat, *P. claussenii* M | 10.1 | 14.7 | 11.2 | 16.4 | 14.0 |
| Wheat, *L. brevis* WT | 11.3 | 14.7 | 10.5 | 16.4 | 13.5 |
| Wheat, *L. brevis* M | 10.6 | 13.7 | 10.5 | 18.9 | 13.1 |
| Rye, *P. claussenii* WT | 10.1 | 11.1 | 8.9 | 20.8 | 14.5 |
| Rye, *P. claussenii* M | 10.7 | 12.3 | 8.8 | 17.2 | 14.6 |
| Rye, *L. brevis* WT | 10.8 | 10.4 | 8.5 | 20.0 | 14.7 |
| Rye, *L. brevis* M | 11.8 | 13.2 | 9.4 | 17.8 | 14.5 |

*Pediococcus (P.) claussenii* (WT: TMW 2.340, M: TMW 2.2123) and *Levilactobacillus (L.) brevis* (WT: TMW 1.2112, M: TMW 1.2320). M: mutant, FP: fermentation pellet, TMW: Technische Mikrobiologie Weihenstephan, WT: wild-type, T0: Feces inoculum (time point before *in vitro* fermentation), (n=1).

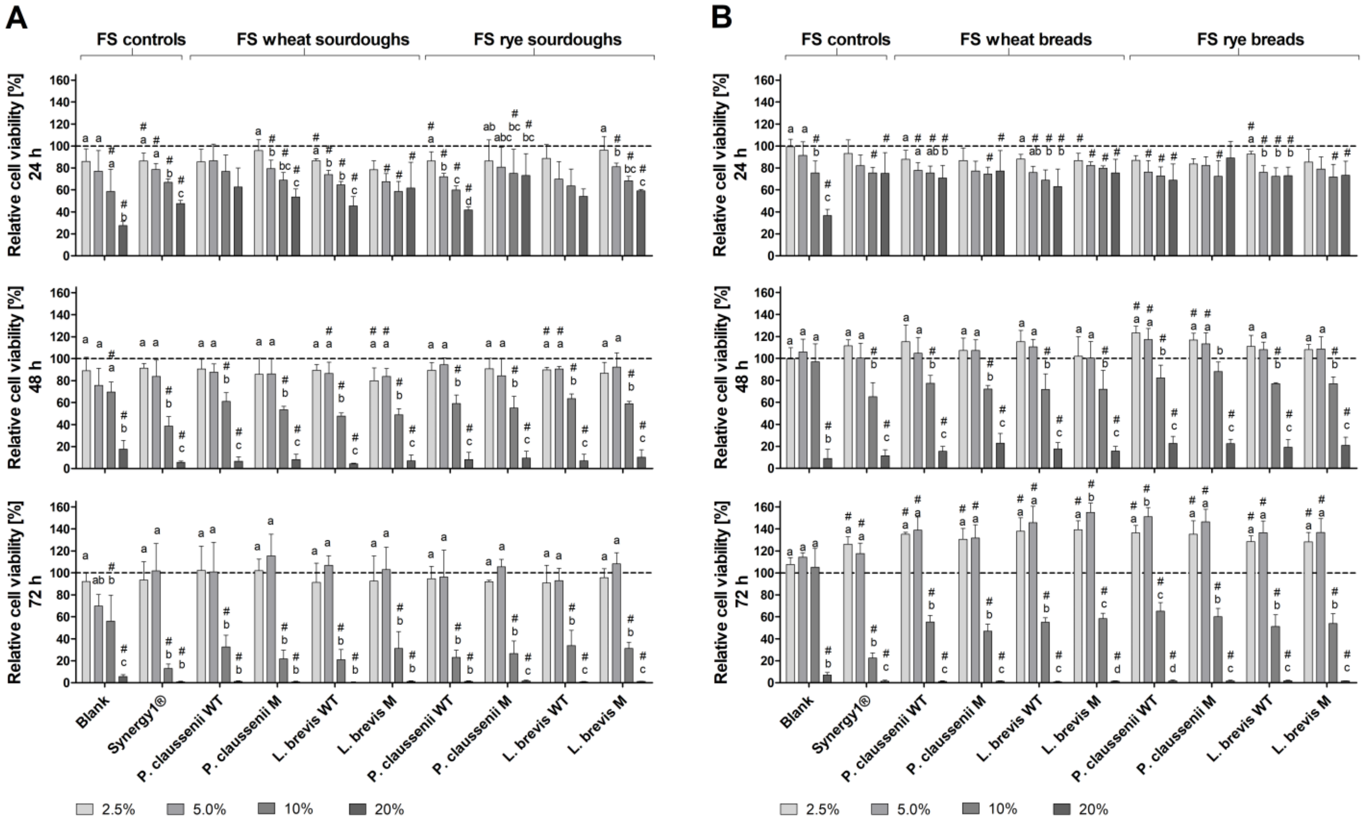


**Supplemental Figure S1.** Relative viability [%] of LT97 colon adenoma cells after treatment with different concentrations (2.5%, 5%, 10% and 20%) of fermentation supernatants (FS) obtained from controls (blank, Synergy1®) and wheat and rye sourdoughs (**A**) and FS obtained from controls (blank, Synergy1®) and wheat and rye breads (**B**) for 24 h, 48 h and 72 h. Sourdoughs and breads were generated using wild-type (WT) and mutant (M) strains of *Pediococcus claussenii* (WT: TMW 2.340, M: TMW 2.2123) and *Levilactobacillus brevis* (WT: TMW 1.2112, M: TMW 1.2320). Results were calculated on the basis of the medium control (dashed line), which was set 100% (mean + SD, n = 3 (A) and n = 4 (B)). Significant differences between cells treated with FS blank and cells treated with FS of all other samples ($^{\#}$ $p < 0.05$) and significant differences between cells treated with different concentrations of FS ($^{a\text{-}c}$ $p < 0.05$, different letters represent significantly different results) were obtained by One-Way ANOVA and F-test according to Ryan-Einot-Gabriel-Welsh. TMW: Technische Mikrobiologie Weihenstephan.

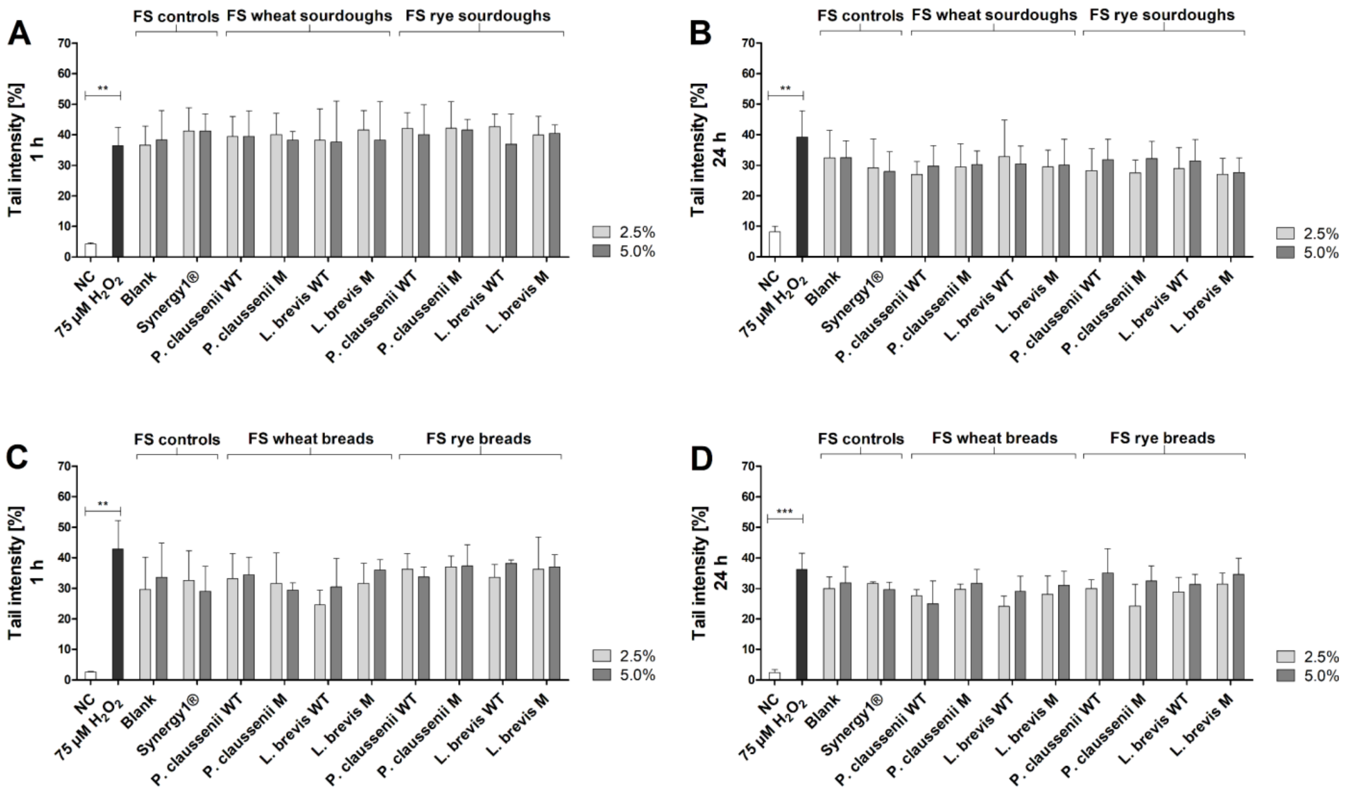


**Supplemental Figure S2.** Tail intensity [%] after treatment of LT97 cells with 2.5% and 5% fermentation supernatants (FS) obtained from controls (blank, Synergy1®) and wheat and rye sourdoughs for 1 h (**A**) and 24 h (**B**) and with FS obtained from controls (blank, Synergy1®) and wheat and rye breads for 1 h (**C**) and 24 h (**D**) (mean + SD, n = 3) after challenging with 75 µM $H_2O_2$ for 15 min. Sourdoughs and breads were generated using wild-type (WT) and mutant (M) strains of *Pediococcus claussenii* (WT: TMW 2.340, M: TMW 2.2123) and *Levilactobacillus brevis* (WT: TMW 1.2112, M: TMW 1.2320). Significant differences between cells treated with FS and negative controls (NC, PBS (1 h)/medium (24 h)) were checked using the One-Way ANOVA. Significant differences between NC and positive control (75 µM $H_2O_2$) were obtained by unpaired Student`s t-test (** $p < 0.01$, *** $p < 0.001$). TMW: Technische Mikrobiologie Weihenstephan.

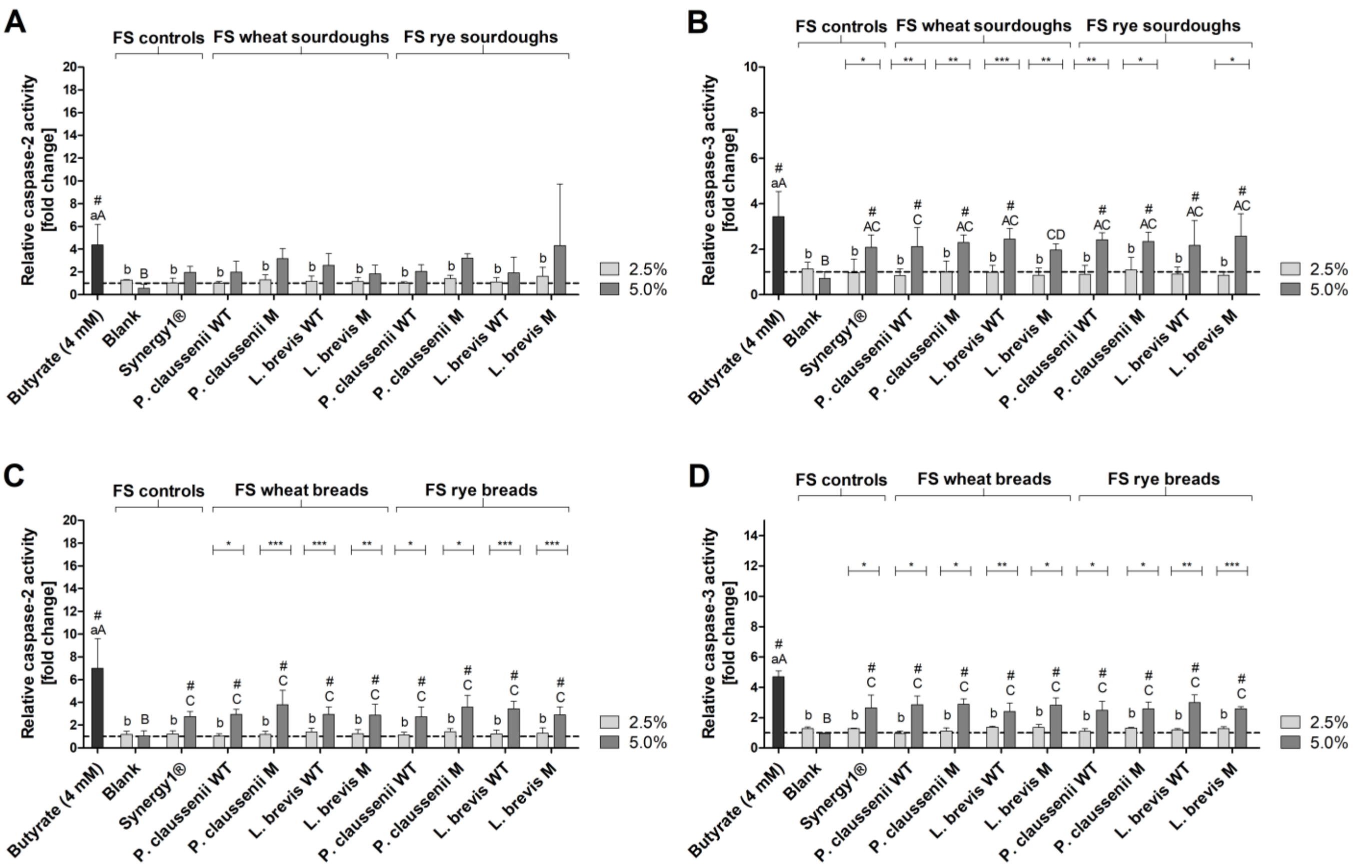


**Supplemental Figure S3.** Relative activities of caspase-2 and caspase-3 in LT97 cells after treatment with 2.5% and 5% fermentation supernatant (FS) obtained from controls (blank, Synergy1®) and wheat and rye sourdoughs (**A, B**) and with FS obtained from controls (blank, Synergy1®) and wheat and rye breads (**C, D**) as well as 4 mM butyrate for 48 h (mean + SD, n = 3). Results are presented as fold changes based on a medium control, which was set 1 (dashed line). Sourdoughs and breads were generated using wild-type (WT) and mutant (M) strains of *Pediococcus claussenii* (WT: TMW 2.340, M: TMW 2.2123) and *Levilactobacillus brevis* (WT: TMW 1.2112, M: TMW 1.2320). Significant differences to the medium control ($^{\#}$ $p < 0.05$) and significant differences between cells treated with 2.5% FS as well as butyrate ($^{a\text{-}b}$ $p < 0.05$) and 5% FS as well as butyrate ($^{A\text{-}D}$ $p < 0.05$) were obtained by Two-Way ANOVA and F-test according to Ryan-Einot-Gabriel-Welsh. Different letters represent significantly different results. Significant differences between cells treated with 2.5% and 5% FS were obtained by unpaired Student's t-test ($^{*}$p < 0.05, $^{**}$p < 0.01, $^{***}$p < 0.001). TMW: Technische Mikrobiologie Weihenstephan.

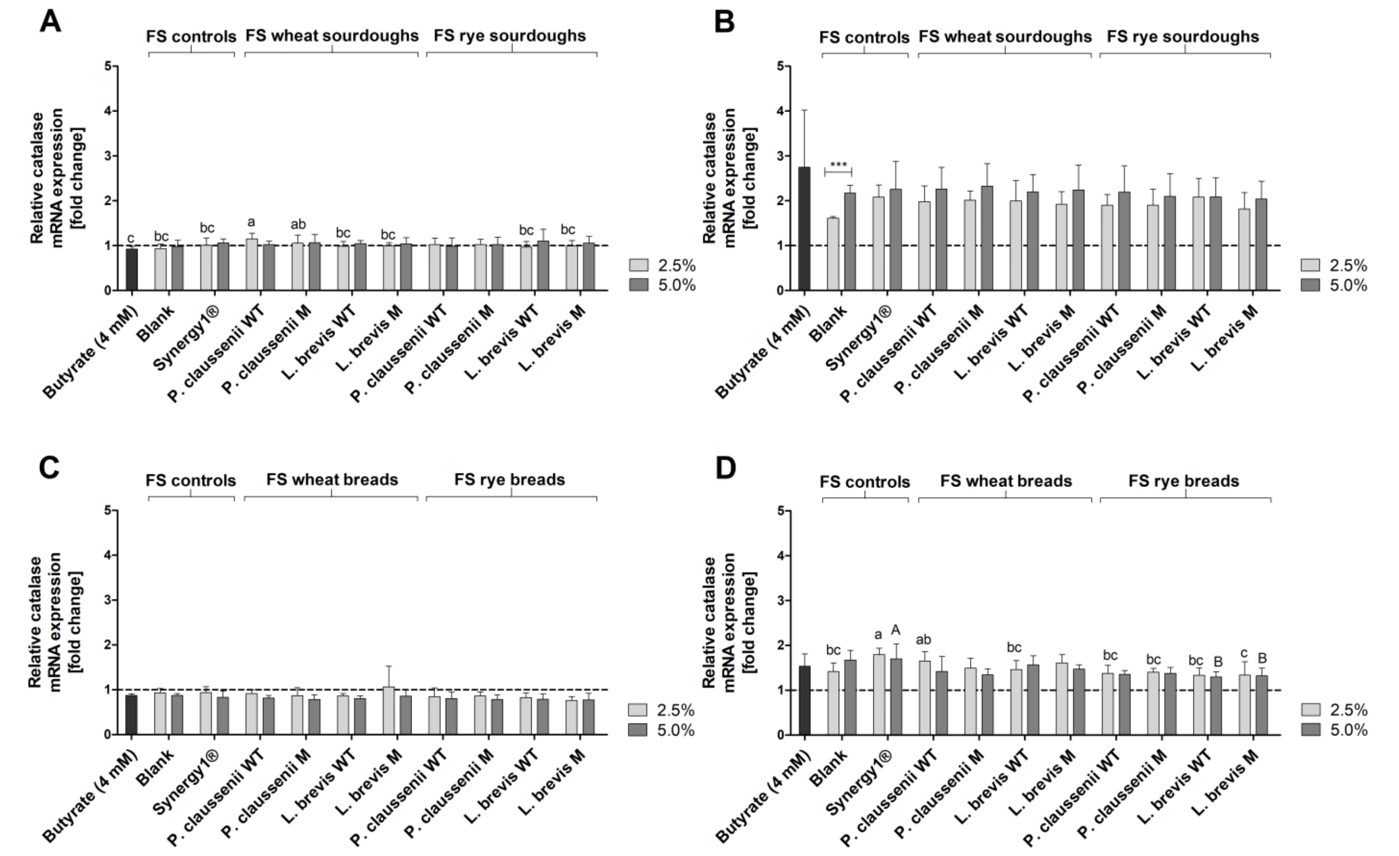


**Supplemental Figure S4.** Relative *catalase* mRNA expression in LT97 cells after treatment with 2.5% and 5% fermentation supernatants (FS) obtained from controls (blank, Synergy1®) and wheat and rye sourdoughs as well as 4 mM butyrate for 6 h (**A**) and 24 h (**B**) and with FS obtained from controls (blank, Synergy1®) and wheat and rye breads as well as 4 mM butyrate for 6 h (**C**) and 24 h (**D**) (mean + SD, n = 4). Results are presented as fold changes based on a medium control, which was set 1 (dashed line). Sourdoughs and breads were generated using wild-type (WT) and mutant (M) strains of *Pediococcus claussenii* (WT: TMW 2.340, M: TMW 2.2123) and *Levilactobacillus brevis* (WT: TMW 1.2112, M: TMW 1.2320). Significant differences between cells treated with 2.5% FS as well as butyrate ([a-b] $p < 0.05$) and 5% FS as well as butyrate ([A-C] $p < 0.05$) were obtained by Two-Way ANOVA and F-test according to Ryan-Einot-Gabriel-Welsh from log-transformed data. Different letters represent significantly different results. Significant differences between cells treated with 2.5% and 5% FS were obtained by unpaired Student's t-test ([*]p < 0.05). TMW: Technische Mikrobiologie Weihenstephan.

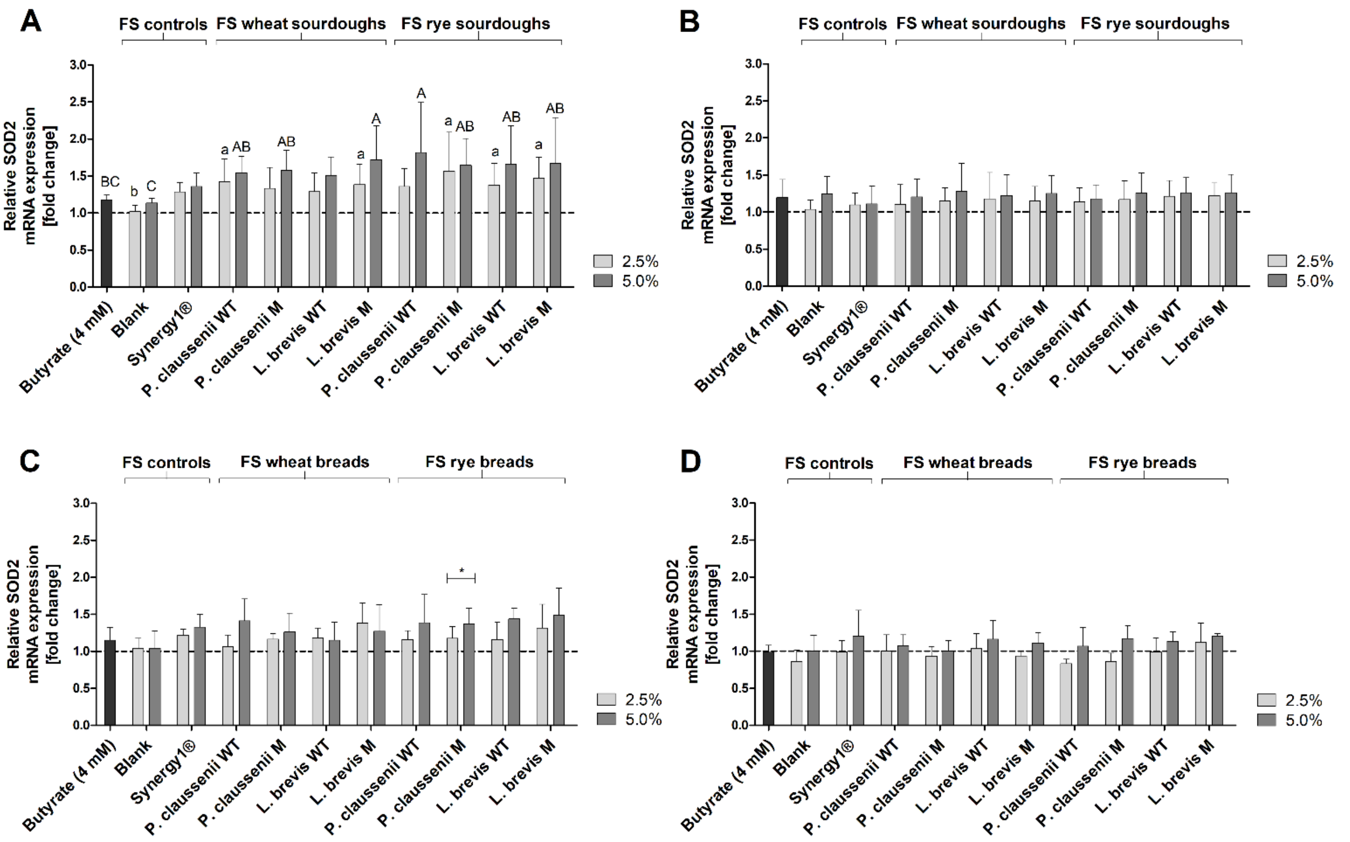


**Supplemental Figure S5.** Relative *SOD2* mRNA expression in LT97 cells after treatment with 2.5% and 5% fermentation supernatants (FS) obtained from controls (blank, Synergy1®) and wheat and rye sourdoughs as well as 4 mM butyrate for 6 h (**A**) and 24 h (**B**) and with FS obtained from controls (blank, Synergy1®) and wheat and rye breads as well as 4 mM butyrate for 6 h (**C**) and 24 h (**D**) (mean + SD, n = 4). Results are presented as fold changes based on a medium control, which was set 1 (dashed line). Sourdoughs and breads were generated using wild-type (WT) and mutant (M) strains of *Pediococcus claussenii* (WT: TMW 2.340, M: TMW 2.2123) and *Levilactobacillus brevis* (WT: TMW 1.2112, M: TMW 1.2320). Significant differences between cells treated with 2.5% FS as well as butyrate ($^{a\text{-}b}$ $p < 0.05$) and 5% FS as well as butyrate ($^{A\text{-}C}$ $p < 0.05$) were obtained by Two-Way ANOVA and F-test according to Ryan-Einot-Gabriel-Welsh from log-transformed data. Different letters represent significantly different results. Significant differences between cells treated with 2.5% and 5% FS were obtained by unpaired Student's t-test ($^{*}p < 0.05$, $^{**}p < 0.01$). TMW: Technische Mikrobiologie Weihenstephan.

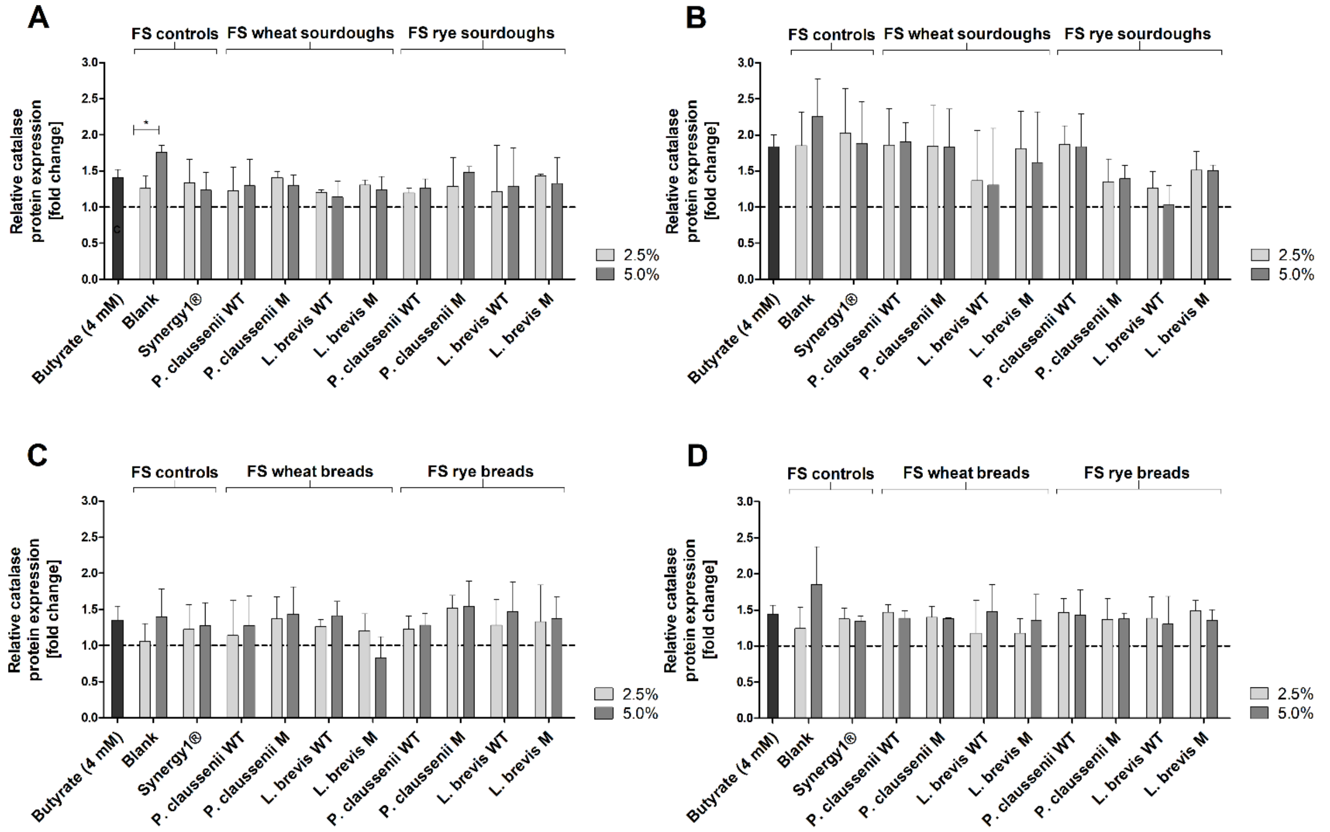


**Supplemental Figure S6.** Relative catalase protein expression in LT97 cells after treatment with 2.5% and 5% fermentation supernatants (FS) obtained from controls (blank, Synergy1®) and wheat and rye sourdoughs as well as 4 mM butyrate for 24 h (**A**) and 48 h (**B**) and with FS obtained from controls (blank, Synergy1®) and wheat and rye breads as well as 4 mM butyrate for 24 h (**C**) and 48 h (**D**) (mean + SD, n = 3). Results are presented as fold changes based on a medium control, which was set 1 (dashed line). Sourdoughs and breads were generated using wild-type (WT) and mutant (M) strains of *Pediococcus claussenii* (WT: TMW 2.340, M: TMW 2.2123) and *Levilactobacillus brevis* (WT: TMW 1.2112, M: TMW 1.2320). Significant differences between cells treated with 2.5% FS as well as butyrate and 5% FS as well as butyrate were checked by One-Way ANOVA and F-test according to Ryan-Einot-Gabriel-Welsh. Significant differences between cells treated with 2.5% and 5% FS were obtained by unpaired Student's t-test (*p < 0.05). TMW: Technische Mikrobiologie Weihenstephan.

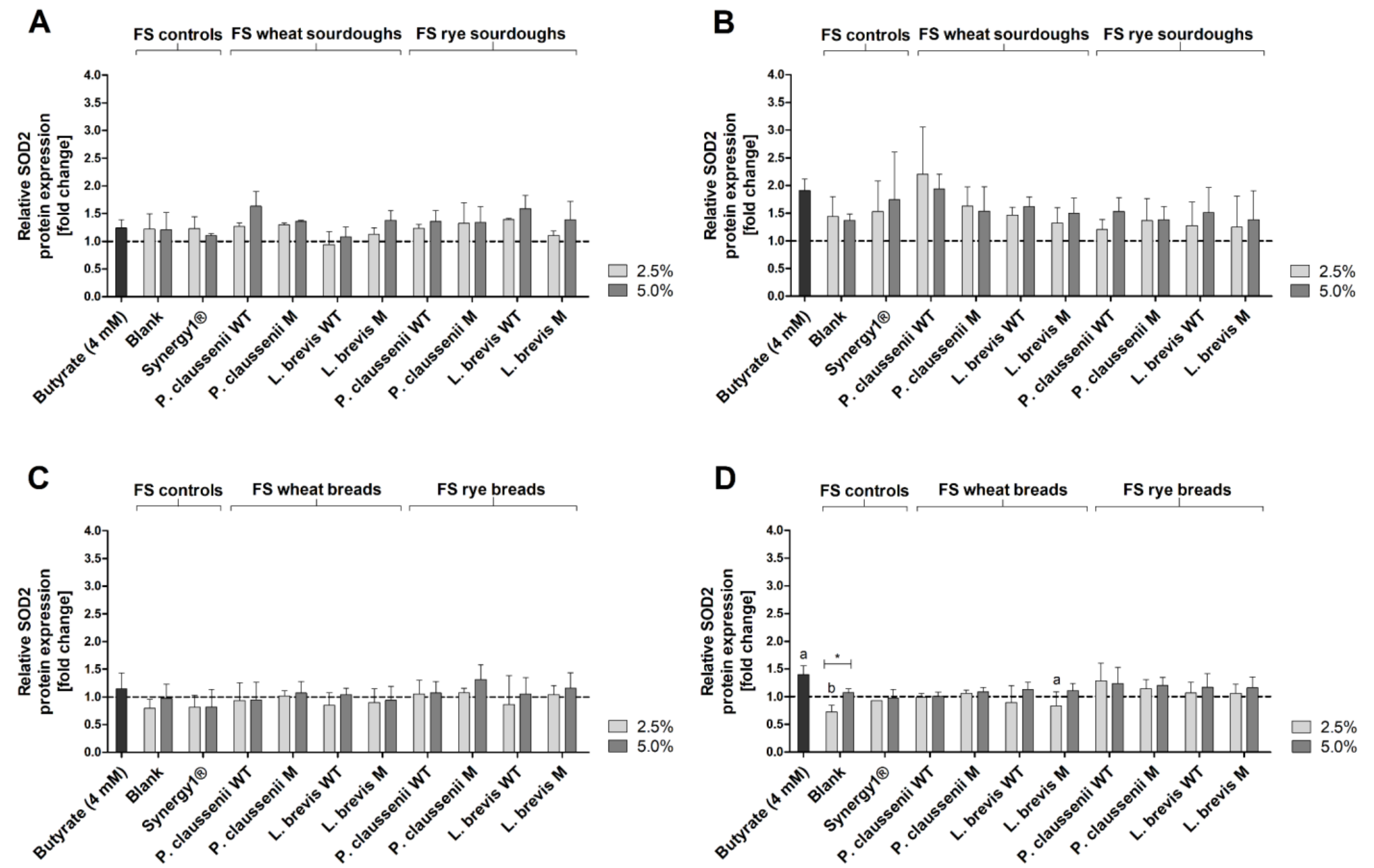


**Supplemental Figure S7.** Relative SOD2 protein expression in LT97 cells after treatment with 2.5% and 5% fermentation supernatants (FS) obtained from controls (blank, Synergy1®) and wheat and rye sourdoughs as well as 4 mM butyrate for 24 h (**A**) and 48 h (**B**) and with FS obtained from controls (blank, Synergy1®) and wheat and rye breads as well as 4 mM butyrate for 24 h (**C**) and 48 h (**D**) (mean + SD, n = 3). Results are presented as fold changes based on a medium control, which was set 1 (dashed line). Sourdoughs and breads were generated using wild-type (WT) and mutant (M) strains of *Pediococcus claussenii* (WT: TMW 2.340, M: TMW 2.2123) and *Levilactobacillus brevis* (WT: TMW 1.2112, M: TMW 1.2320). Significant differences between cells treated with 2.5% FS as well as butyrate ($^{a\text{-}b}$ $p < 0.05$) and 5% FS as well as butyrate were obtained by One-Way ANOVA and F-test according to Ryan-Einot-Gabriel-Welsh. Different letters represent significantly different results. Significant differences between cells treated with 2.5% and 5% FS were obtained by unpaired Student's t-test ($^*p < 0.05$). TMW: Technische Mikrobiologie Weihenstephan.

Protein expression: p21, GAPDH in LT97 cells after treatment with FS of sourdoughs for 24 h:

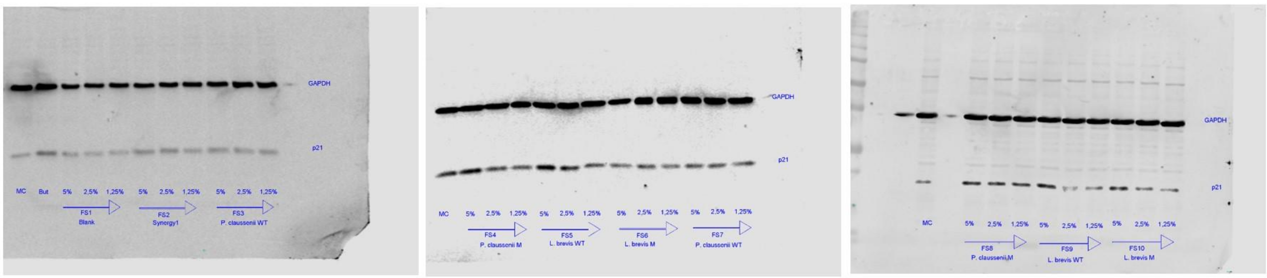


Protein expression: p21, GAPDH in LT97 cells after treatment with FS of sourdoughs for 48 h:

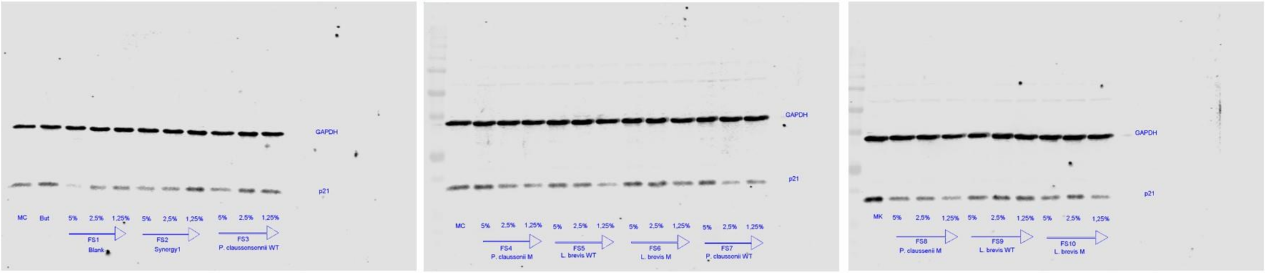


Protein expression: Cyclin D2, GAPDH in LT97 cells after treatment with FS of sourdoughs for 24 h:

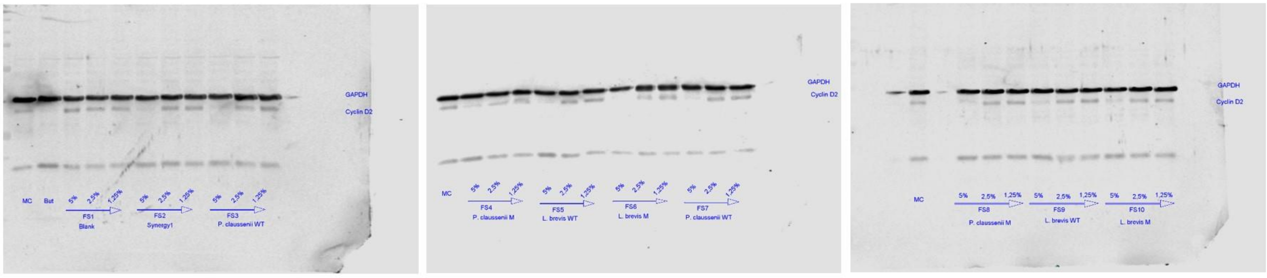


Protein expression: Cyclin D2, GAPDH in LT97 cells after treatment with FS of sourdoughs for 48 h:

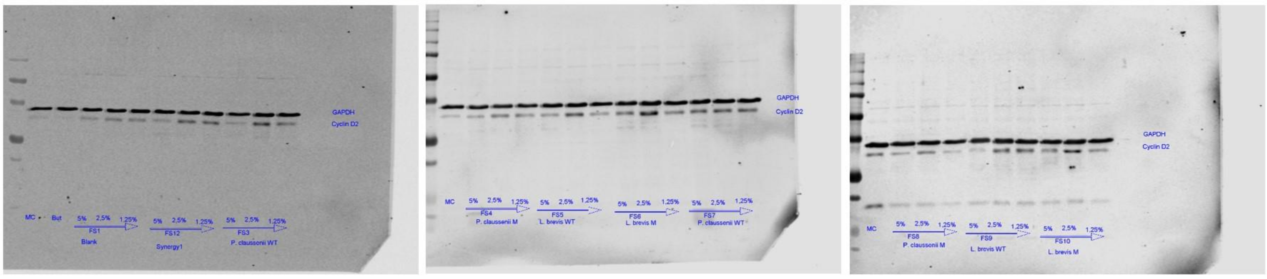


Protein expression: Catalase, SOD2, GAPDH in LT97 cells after treatment with FS of sourdoughs for 24 h:

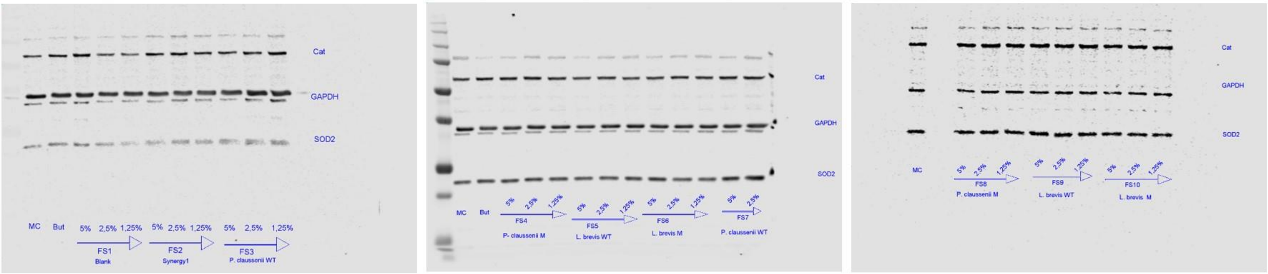


Protein expression: Catalase, SOD2, GAPDH in LT97 cells after treatment with FS of sourdoughs for 48 h:

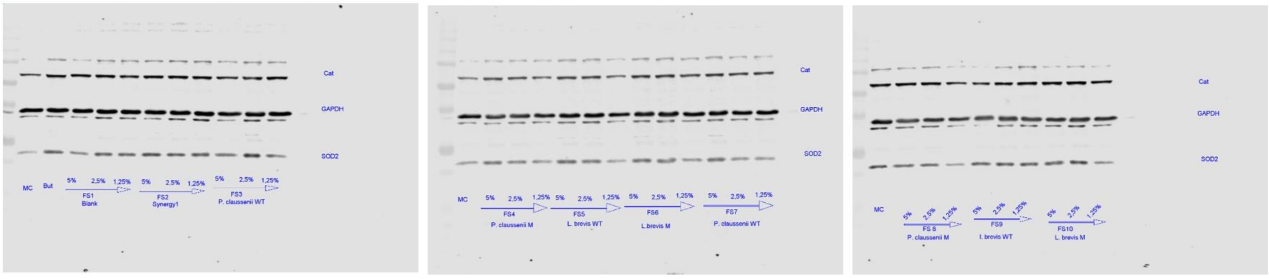


**Supplemental Figure S8.** Representative images of western blot analyses used for protein expression analyses of p21, cyclin D2, catalase (Cat), SOD2 and GAPDH (reference protein) in LT97 cells after treatment with fermentation supernatants (FS) obtained from wheat and rye sourdoughs generated with wild-type (WT) and mutant (M) strains of *Pediococcus claussenii* (WT: TMW 2.340, M: TMW 2.2123) and *Levilactobacillus brevis* (WT: TMW 1.2112, M: TMW 1.2320). TMW: Technische Mikrobiologie Weihenstephan.

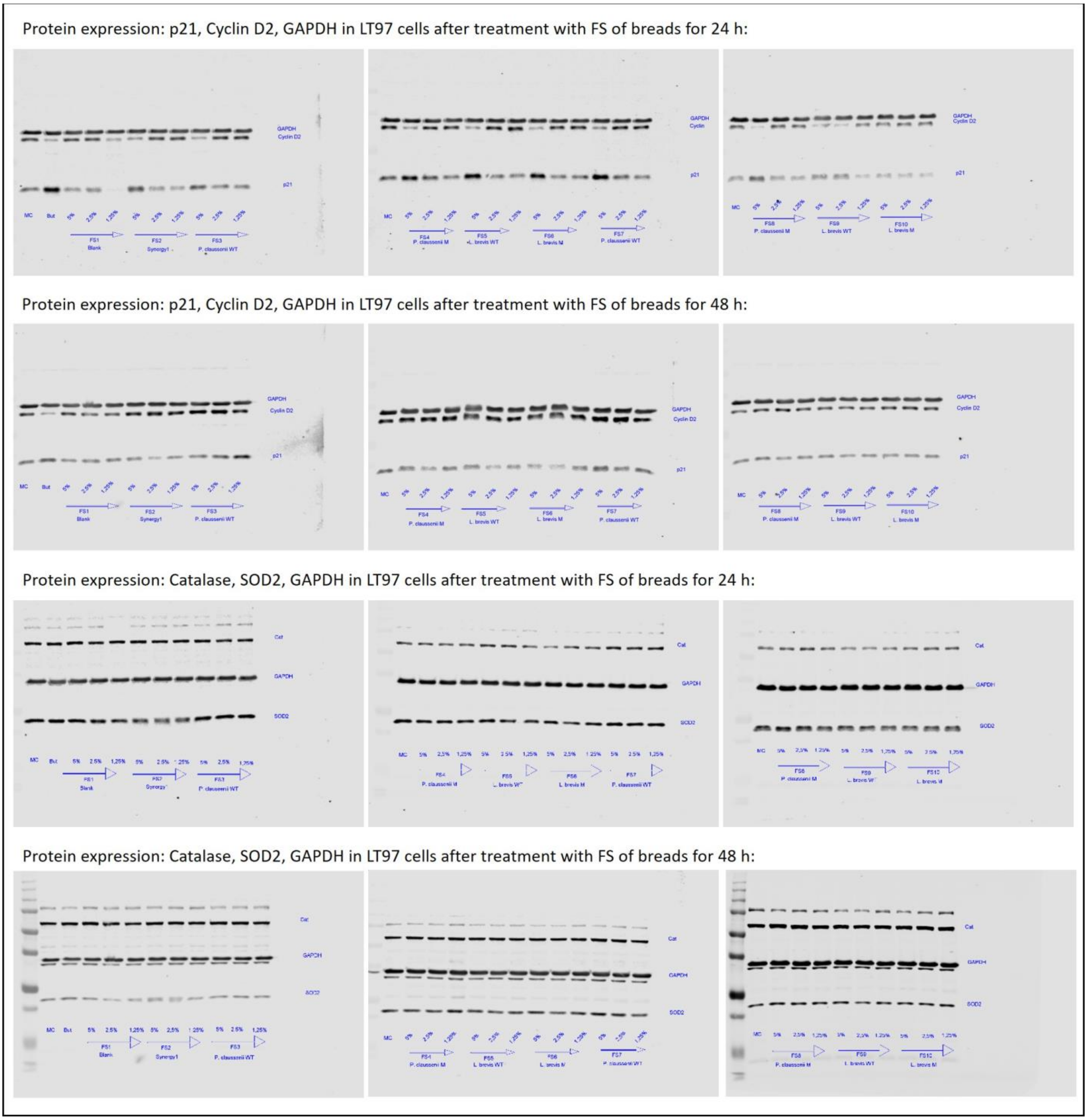


**Supplemental Figure S9.** Representative images of western blot analyses used for protein expression analyses of p21, cyclin D2, catalase (Cat), SOD2 and GAPDH (reference protein) in LT97 cells after treatment with fermentation supernatants (FS) obtained from wheat and rye breads generated with wild-type (WT) and mutant (M) strains of *Pediococcus claussenii* (WT: TMW 2.340, M: TMW 2.2123) and *Levilactobacillus brevis* (WT: TMW 1.2112, M: TMW 1.2320). TMW: Technische Mikrobiologie Weihenstephan.
